# Organic–inorganic hybrid salt and mixed ligand Cr(III) complexes containing the natural flavonoid chrysin: Synthesis, characterization, computational, and biological studies

**DOI:** 10.3389/fchem.2023.1173604

**Published:** 2023-04-12

**Authors:** Mamaru Bitew Alem, Tegene Desalegn, Tadewos Damena, Enyew Alemayehu Bayle, Moses O. Koobotse, Kennedy J. Ngwira, Japheth O. Ombito, Matshediso Zachariah, Taye B. Demissie

**Affiliations:** ^1^ Department of Applied Chemistry, Adama Science and Technology University, Adama, Ethiopia; ^2^ Department of Chemistry, Wachemo University, Hossana, Ethiopia; ^3^ Graduate Institute of Applied Science and Technology, National Taiwan University of Science and Technology, Taipei, Taiwan; ^4^ Department of Chemistry, Debre Markos University, Debre Markos, Ethiopia; ^5^ School of Allied Health Professions, University of Botswana, Gaborone, Botswana; ^6^ Molecular Sciences Institute, School of Chemistry, University of the Witwatersrand, Johannesburg, South Africa; ^7^ Department of Chemistry, University of Botswana, Gaborone, Botswana

**Keywords:** chrysin, Cr(III) complex, cytotoxicity, organic–inorganic hybrid salt, molecular docking, MCF-7

## Abstract

Organic–inorganic hybrid salt and mixed ligand Cr(III) complexes (**Cr1** and **Cr2**) containing the natural flavonoid chrysin were synthesized. The metal complexes were characterized using UV-Vis, Fourier-transform infrared, MS, SEM-EDX, XRD, and molar conductance measurements. Based on experimental and DFT/TD-DFT calculations, octahedral geometries for the synthesized complexes were suggested. The powder XRD analysis confirms that the synthesized complexes were polycrystalline, with orthorhombic and monoclinic crystal systems having average crystallite sizes of 21.453 and 19.600 nm, percent crystallinities of 51% and 31.37%, and dislocation densities of 2.324 × 10^−3^ and 2.603 × 10^−3^ nm^-2^ for **Cr1** and **Cr2**, respectively. The complexes were subjected to cytotoxicity, antibacterial, and antioxidant studies. The *in vitro* biological studies were supported with quantum chemical and molecular docking computational studies. **Cr1** showed significant cytotoxicity to the MCF-7 cell line, with an IC_50_ value of 8.08 μM compared to 30.85 μM for **Cr2** and 18.62 μM for cisplatin. **Cr2** showed better antibacterial activity than **Cr1**. The higher *E*_HOMO_ (−5.959 eV) and dipole moment (10.838 Debye) values of **Cr2** obtained from the quantum chemical calculations support the observed *in vitro* antibacterial activities. The overall results indicated that **Cr1** is a promising cytotoxic drug candidate.

## 1 Introduction

Coordination compounds (CCs) have a more important place in chemistry as functional materials owing to their exceptional structural arrangements (Kilpin and Dyson, 2013; Renfrew, 2014). CCs have a wide range of applications, especially in medicine (diagnosis and treatment), biology, and therapeutics (Renfrew, 2014; Kasprzak et al., 2015; Boros et al., 2020; Karges et al., 2021; Damena et al., 2022; Olelewe and Awuah, 2023). As a result, interest in metal-based therapeutic agents is growing in the bioinorganic chemistry era due to the emergence of clinical trials with various mechanisms of action that could potentially provide new chemotherapeutic approaches to disease management (Boros et al., 2020; Damena et al., 2022; Scalese et al., 2022; Kacsir et al., 2023; Olelewe and Awuah, 2023). Numerous metal-based promising chemotherapeutic agents have been reported since the discovery of cisplatin for general oncology in 1978 (Kilpin and Dyson, 2013; Renfrew, 2014; Karges et al., 2021). However, anticancer agents from the first d-block elements series and essential abundant elements are not well explored (Chen et al., 2021). Chromium is among the ubiquitous metals considered to be biologically relevant (Vincent, 2004), both in animal feeding and human nutrition in its trivalent state (Dong et al., 2018). Chromium is believed to be involved in the metabolism of carbohydrates, lipids, and proteins mainly by increasing the efficiency of insulin (Vincent, 2004; Pechova and Pavlata, 2007; Kralovec et al., 2009).

Following the first suggestion that Cr(III) compounds could act as a nutritional enhancement to glucose metabolism in 1959 by Schwarz and Mertz (Pechova and Pavlata, 2007), there has been a search for Cr(III) supplements, with Cr(III)-picolinate being the oldest. Furthermore, *in vitro* and *in vivo* findings have shown that trivalent chromium, Cr(III), has biological benefits, such as enhancing the antioxidant enzyme defense system and facilitating insulin signaling or sensitivity (Anderson et al., 1997; Hao et al., 2011), suppressing free radical formation and inhibiting protein glycosylation (Dong et al., 2018), decreasing serum glucose levels (Pandey et al., 2012), lowering blood lipid levels (Anderson et al., 1997), reducing cholesterol and triglyceride levels in the blood (Wang et al., 2017), lowering glycated hemoglobin levels (El-Megharbel, 2015), and inhibiting α-glucosidase activity (Dong et al., 2018). However, the bioavailability and efficacy of Cr(III) are dependent on the ligands that chelate to chromium ions (Peng and Yang, 2015; Ulas et al., 2015) as the specific ligand environment around Cr(III) could affect the biological activity and bioavailability of chromium (Ahmad et al., 2012; Peng and Yang, 2015). However, organic–inorganic hybrid salts of chromium show promising applications as functional materials in various fields, such as catalysis, magnetism, medicine, cancer therapy, luminescence, conductivity, material science, and industrial applications (Ndong et al., 2020; Zare et al., 2021).

In this work, we designed an organic–inorganic hybrid salt consisting of a natural flavonoid chrysin and dichlorobis (1,10-phenanthroline)chromate (III) as Lewis base and acid, respectively, where CrCl_3_.6H_2_O salt was used as a metal precursor. Moreover, metformin-coordinated Cr(III) complexes reportedly exhibit antibacterial activity and cytotoxicity. We previously reported the synthesis, antibacterial, and cytotoxicity profiles of Cu(II) complexes using 1,10-phenanthroline and the drugs metformin and ciprofloxacin (Alem et al., 2022), which showed the promising cytotoxicity profiles of Cu(II) mixed drug complexes against the MCF-7 human breast cancer cell line. Inspired by the previous findings and due to our interest in examining the innate cytotoxicity of Cr(III) against MCF-7, we synthesized and evaluated the biological activities of two metal complexes of Cr(III) using 1,10-phenanthroline, metformin, and chrysin ligands.

## 2 Materials and methods

### 2.1 Materials

All chemicals and reagents were of analytical grade and used without any treatment. 1,10-phenanthroline (BDH chemical Ltd., Poole, England), chrysin (Sigma Aldrich), chromium chloride hexahydrate (CrCl_6_.6H_2_O), dimethyl sulfoxide (DMSO), and dimethylformamide (DMF) were purchased from Loba Chemie Pvt. Ltd. (Mumbai, India). Triethylamine, NaHCO_3_, Mueller–Hinton agar, methanol (MeOH), and diethyl ether were purchased from (Alpha Chemika, India). Ascorbic acid and 2,2-diphenyl-1-picrylhydrazyl (DPPH) were purchased from Sigma-Aldrich.

### 2.2 Synthesis

#### 2.2.1 Synthesis of organic–inorganic hybrid salts of Cr(III)

A heteroleptic Cr(III)complex (**Cr1**) of 1,10-phenanthroline and an oxo ligand, chrysin (5,7-dihydroxy-2-phenyl-4H-chromen-4-one) was prepared as reported previously by Abebe and Hailemariam (2016) with minor modifications. In brief, 1 mmol of chrysin (0.254 g) was added to a mixture of 20 mL of methanol:ethanol solution (1:3 ratio) in the presence of a deprotonating agent, 1 mmol NaOH, and stirred for 20 min, resulting in a clear yellow solution. To this solution, 1 mmol of CrCl_3_.6H_2_O (0.266 g) was added slowly and refluxed at 90°C in an oil bath. The resulting light green solution was refluxed for 1 h 20 min; then, 1 mmol of methanolic solution of 1,10-phenanthroline was added slowly. A drop of triethylamine was added to the reaction mixture to maintain the alkaline pH of the solution and the reflux was continued for an additional 6 h 40 min. The resulting brown solution was filtered and cooled. Next, 10 mL of diethyl ether was added to the filtrate and allowed to stand overnight. The resulting jelly-like brown precipitate was left to dry slowly at room temperature.

**SCHEME 1:**
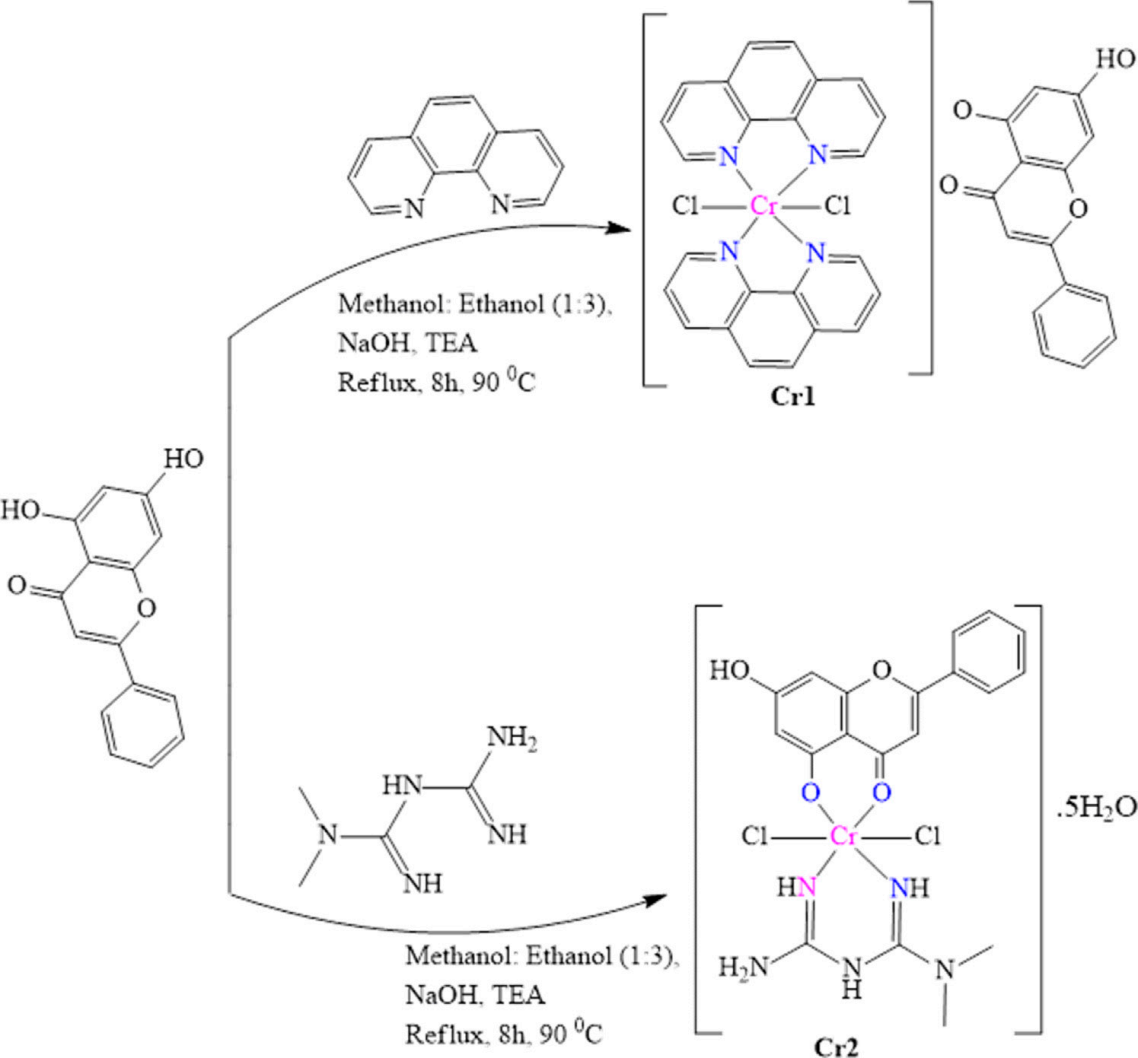
Schematic representation of the synthesis of **Cr(III)** complexes.

For **Cr2,** which consisted of metformin and oxo ligand, chrysin was prepared as reported by Lin et al. (Abd El-Halim et al., 2017) with minor modifications. Briefly, to the clear hot methanolic solution of metformin, 1 mmol of CrCl_3_.6H_2_O (0.266 g) was added slowly and stirred, resulting in a faded green solution. To the reaction mixture, 1 mmol of chrysin (0.254 g) dissolved in a mixture of methanol:ethanol (1:3) was added in the presence of a deprotonating agent, 1 mmol NaOH. Triethylamine (1 mmol, 140 μL) was used to maintain the alkaline pH of the solution and the reaction was refluxed for an additional 8 h. The resulting greenish-brown solution was filtered, treated with diethyl ether, and allowed to stand for 2 weeks. A light green powder was obtained after the slow evaporation at room temperature. The overall synthesis scheme is presented in Scheme 1.

### 2.3 Instrumentation

Fourier-transform infrared (FTIR) spectroscopy was recorded from 4,000 to 400 cm^−1^ in KBr pellets on a PerkinElmer BX FTIR spectrometer. The UV-Vis spectra were recorded from 200–800 nm on an SM-1600 spectrophotometer using dilute solutions (1.0 × 10^−4^ M) of the test compounds. The thermogravimetric/differential thermal analyses (TGA/DTA) data were recorded from 25 to 800°C at a heating rate of 10^°^C/min under nitrogen-atmosphere (20 mL/min) on a Shimadzu DTG-60H thermal analyzer. X-ray diffraction (XRD-7000, Shimadzu Co., Japan) was used to determine the crystalline nature of the synthesized metal complexes. The diffraction was recorded with 2*θ* = 10 to 80 using CuK*α* (*λ* = 1*.*5406 Å) radiation at 40 kV and 30 mA (Holzwarth and Gibson, 2011). The inter-planar spacing (*d*), Miller indices (*hkl*), and lattice parameters (a, b, c, α, β, and γ) were estimated using QUALX2.0 (Altomare et al., 2015). SEM-EDX measurements were performed with a Joel JSM-6500F instrument (Joel, Tokyo, Japan). The high-resolution mass spectra were obtained with a Waters LCT Premier mass spectrometer using a sample concentration of 2 ng/μL with a capillary voltage of 2500 V and a desolvation temperature of 250°C using nitrogen gas at 250 L/h, Bruker APEX II CCD area detector diffractometer, with graphite monochromated Mo K3 radiation (50 kV, 30 mA) and temperature of measurement at 1732 K coupled with APEX 2 data collection software.

### 2.4 Biological activity studies

#### 2.4.1 Cytotoxicity evaluation against the MCF-7 cell line

The cytotoxicities of the complexes (**Cr1** and **Cr2**) were evaluated using the 3-[4,5-dimethylthiazole-2-yl]-2,5-diphenyltetrazolium bromide (MTT) assay. The detailed cell culture conditions and procedure for the cytotoxicity assay were reported previously (Alem et al., 2022).

#### 2.4.2 Antibacterial activity

The *in vitro* antibacterial activities of the metal complexes were evaluated against the Gram-positive (*Staphylococcus aureus* (ATCC25923) and *Streptococcus* pyogenes (ATCC19615)) and Gram-negative (*Escherichia coli* (ATCC25922)) bacterial strains and *Pseudomonas aeruginosa* (ATCC27853) using the disk diffusion method in Mueller–Hinton agar (MHA) (Ommenya et al., 2020). All protocols and the negative and positive controls were similar to those reported previously (Lemilemu et al., 2021). Known concentrations (500 and 1,000 μM) of the metal complexes were prepared in DMSO. The analysis was conducted in triplicate, and the results were presented as a mean ± SD.

#### 2.4.3 DPPH radical scavenging activity

The DPPH scavenging activities of the synthesized complexes (**Cr1** and **Cr2**) were evaluated as we described previously, with minor modifications (Lemilemu et al., 2021; Alem et al., 2022; Damena et al., 2023). The percent DPPH free radical scavenging activities of the synthesized complexes and the standard were calculated using Equation (1).

$$\%\;DPPH\;radical\;scavenging\;activity=\frac{Ac-As}{Ac}\times 100\%,$$ (1)

where A_c_ is the absorbance of the control and A_s_ is the absorbance of the sample.

### 2.5 Quantum chemical studies

Density functional theory (DFT) employing the B3LYP (Lee et al., 1988; Becke, 1993; Stephens et al., 1994) hybrid functional together with the 6–311++G (d, p) basis set (Krishnan et al., 1980) for atoms of the ligand and the Los Alamos National Laboratory 2-double-zeta (LanL2DZ) pseudopotential for the chromium atom (Hay and Wadt, 1985) were employed, respectively. Grimme’s dispersion correction (Grimme, 2004) was employed to account for non-bonding interactions. Vibrational frequency calculations were used to ensure that the optimized geometries were real minima without imaginary frequencies. The time-dependent DFT (TD-DFT) was used to calculate the absorption energies. The calculated absorption spectra were red-shifted by 30 nm for better comparison with the corresponding experimental spectra. Quantum chemical descriptors calculation, frontier molecular orbital (FMO) visualization, and associated data were conducted as we previously reported (Alem et al., 2022; Damena et al., 2023).

### 2.6 Molecular docking studies

The molecular docking study against estrogen receptor alpha (ERα; PDB: 5G4) (Xie et al., 2017), *S. aureus* (PDB: 2w9h) (Heaslet et al., 2009), and *E. coli* (PDB: 6F86) (Narramore et al., 2019) proteins were performed using AutoDock 4.2.6 (Allouche, 2012). Docking protocols similar to our previous works (Bitew et al., 2021; Lemilemu et al., 2021; Alem et al., 2022) were used. Finally, the conformers with the lowest binding free energies were used to visualize the interactions between the active amino acids and the molecules using Discovery Studio software. Cisplatin and ciprofloxacin were used as positive controls to compare the docking results with the corresponding experimental results.

## 3 Results and discussion

### 3.1 Physicochemical properties

The designed complexes (Scheme 1) were synthesized at high yield with corresponding colors of brown and light green for **Cr1** and **Cr2**, respectively (Table 1). Solvents of varying polarity were used to evaluate the solubilities of the synthesized metal complexes. At room temperature, both complexes were soluble in the DMSO and DMF polar aprotic solvents, mildly soluble in the polar water solvents methanol and ethanol, and insoluble in dichloromethane, ethyl acetate, chloroform, and hexane.

**TABLE 1:** Physicochemical properties of the synthesized complexes.

Compound	Color	State	Mass (% yield)	M. pt (°C)	Conductivity (Ω^−1^mol^−1^cm^2^, 25 °C)
**Cr1**	Brown	Powder	0.584 g (83.908)	200–202	53.90
**Cr2**	Light green	Powder	0.484 g (81.344)	>220	104.40

### 3.2 Molar conductance analysis

**SCHEME 2:**
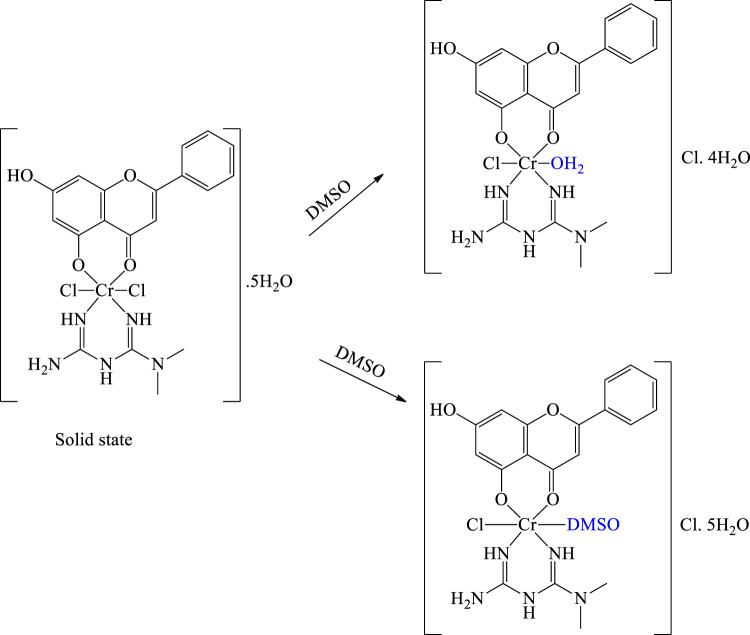
Proposed structures of the solid and solution forms of **Cr2**.

Molar conductance analysis was performed to ascertain the electrolytic and non-electrolytic natures of the synthesized metal complexes. Their molar conductances for **Cr1** and **Cr2** were 53.90 and 104.40 Ω^−1^ mol^−1^ cm^2^, respectively (Table 1). The results indicated that the 1:1 coordination sphere cation and anion ratio of **Cr1** had an electrolytic (ionic) nature (Ali et al., 2013). The higher molar conductance value for **Cr2** could be associated with the presence of water of crystallization and substitution of the two chlorides by the solvent molecules (Alem et al., 2022) (Scheme 2). To confirm the presence/absence of chloride ions in the ionization spheres of the **Cr1** and **Cr2** complexes, a chloride test was performed using 1 mol of AgNO_3_ solution. A negative result for the expected white precipitate was obtained, inferring the absence of chloride ions in the ionization sphere. Hence, the geometries were proposed to be octahedral with molecular formulas of [Cr(C_12_H_8_N_2_)_2_Cl_2_](C_15_H_9_O_4_) and [C_19_H_20_Cl_2_CuN_5_O_4_].5H_2_O, for **Cr1** and **Cr2**, respectively.

### 3.3 FTIR analysis

The FTIR experimental spectra of free chrysin showed characteristic vibrational peaks at 3,439–3,406 cm^-1^, which were computationally identified as 3,698 cm^-1^ and 3,709 cm^-1^ for the fifth and seventh *ν*-OH of chrysin, respectively. Upon coordination with the Cr(III) center, the *ν*-OH vibration appeared at 3,399 and 3,404 cm^-1^ for **Cr1** and **Cr2**, respectively (Supplementary Figures S1, S2). The intensities of *ν*-OH of chrysin in **Cr1** (87% transmittance) and **Cr2** (85% transmittance) were relatively large compared to that for free chrysin (52%). This confirmed that only one of the hydroxyl groups participated in the bonding. Furthermore, the free chrysin vibrations (experimental/calculated) observed at 1,640/1,608 and 2,930/3,123 cm^-1^ occurred due to *ν*-C=O and *ν*-C-H stretching, respectively, similar to previous reports (Sundaraganesan et al., 2012). The *ν*-C=O vibrations shifted to 1,623 cm^-1^ for **Cr1** and 1,655 cm^-1^ for **Cr2**, confirming the change in free chrysin electron conjugation. The large vibrational frequency difference (Δ ∼ 300 cm^-1^) between the experimental and B3LYP calculated *ν*-OH could be due to the presence of an intermolecular hydrogen bond in the experimental measurement. In addition, the experimental/computational stretching vibrational bands of the 1,10-phenanthroline monohydrate ligand for *ν*-C=C (1,622/1,592 cm^-1^) and *ν*-C=N (1,586/1,550 cm^-1^) functional groups shifted to 1,630/1,608 and 1,521/1,429 cm^-1^, respectively, in **Cr1,** with a decrease in intensity. The stretching vibrational bands of the *ν*-OH (3,430 cm^-1^) associated with the water of crystallization of 1,10-phenanthroline were diminished following the metal-ligand coordination. The decreased intensity indicated the formation of a rigid and symmetric structure with the metal center (Abebe et al., 2020). In addition to the observed vibration frequency shifts, the formation of metal–ligand bonds was confirmed by the appearance of new vibrational bands (experimental/calculated) at 649/720 cm^-1^ for Cr-N bonds.

Metformin can be mainly characterized by its N–H stretching vibration, asymmetrical and symmetrical C-N vibrations, and NH_2_ deformation, respectively, from 3,100 to 3,490, at 1,583–1,626, and 1,470–1,540 cm^-1^ in addition to other stretching and bending vibrations (Nakamoto, 2009; Villamizar-Delgado et al., 2020). In this study, these vibrational frequencies appeared at 3,429, 1,637, and 1,483–1,409 cm^-1^. The participation of the nitrogen atom in the coordination with the metal center (**Cr2**) was evidenced by the changes in the intensity and vibrational frequency of the free metformin (Supplementary Figure S2). The formation of metal-ligand bonds was confirmed by the presence of peaks at 750 and 609 cm^-1^, which corresponded to the formation of Cr–O and Cr–N bonds, respectively (Abbas et al., 2019).

### 3.4 UV-Vis analysis

**FIGURE 1:**
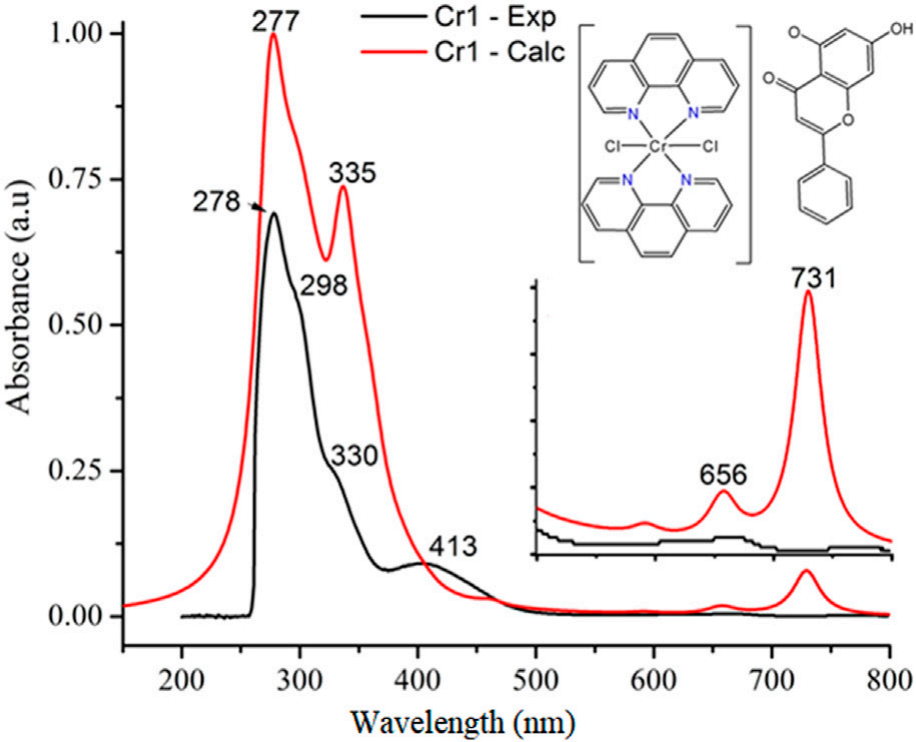
Experimental- (black) and TD-DFT-calculated (red) UV-Vis absorption spectra of **Cr1**.

**FIGURE 2:**
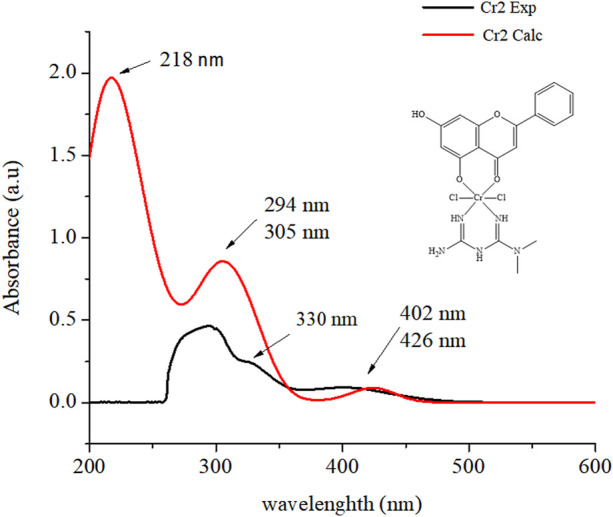
Experimental- (black) and TD-DFT-calculated (red) UV-Vis absorption spectrum of **Cr2**.

The reported UV-Vis of chrysin spectra appear at 270 nm (intense) and 330 nm (less intense), which are associated with the benzoyl system and cinnamoyl system in DMSO (Abd El-Halim et al., 2017). In this study, the UV-Vis/TD-DFT calculated absorption spectra of chrysin in DMSO appeared at 267/261 nm and 320/325 nm for π→π* transitions of the benzoyl and cinnamoyl systems, respectively, consistent with previous reports (Sundaraganesan et al., 2012; Halevas et al., 2021). The absorption spectra of the free ligand 1,10-phenanthroline (experimental/TD-DFT) showed absorption peaks at 228/226 nm and 263/258 nm, corresponding to n→π* (C=N) and π→π*(C=C) transitions, respectively (Tamiru et al., 2019; Alem et al., 2022). The obtained UV-Vis absorption spectrum of the ligands chrysin and 1,10-phenanthroline, and their metal complexes in DMSO together with their TD-DFT calculated results, are presented in Figure 1; Figure 2 and Supplementary Figures S3–S5 in the Supporting Material.

Following the reaction of Cr(III) with the ligands 1,10-phenanthroline and chrysin, bathochromic shifts to the benzoyl and cinnamoyl bands of chrysin were observed, whereas the bands of 1,10-phenanthroline were observed at a longer wavelength (278 nm). The broad peak of chrysin that appeared at 320/325 nm diminished with a slight bathochromic shift (330/335 nm for **Cr1** and 330 nm for **Cr2**), possibly due to the extension of the conjugated system due to complexation. New stronger absorption peaks appearing at 413 nm for **Cr1** (Figure 1) and at 404 nm for **Cr2** (Figure 2 and Supplementary Figure S5) were associated with ligand–ligand charge transfer (LLCT); i.e., the charge transfer between the ligands *via* a Cr(III) metal center (Vogler and Kunkely, 2007).

### 3.5 Mass spectrometric analysis

The mass spectrum of **Cr1** exhibited a peak at *m/z* = 738.109 to 741.114 (found = 735.070), which was attributed to the complex cation [C_39_H_25_Cl_2_CrN_4_O_4_]^+^, and a base peak at *m/z* = 516.077 (found = 514.040) associated with the coordination sphere and methanol (M^+^ + CH_3_OH], whereas a peak at *m/z* = 482.015 (found = 482.020) corresponded to the chemical entities in the coordination sphere, the Cr-metal center, two 1,10-phenanthroline and two chlorides [M = C_24_H_16_Cl_2_CrN_4_]^+^, and an *m/z* value of 255.0653 (found = 254.060) corresponding to the ligand in the ionization sphere of the complex, which is a chrysin fragment [C_15_H_10_O_4_]. The presence of *m*+4 peaks (peaks observed around *m/z* = 482.015 and 516.077) in the mass spectrum confirmed the coordination of two chlorides to the Cr-metal center. In **Cr2**, a molecular ion peak at *m/z* = 595.000 (found = 594.080) attributed to the complex [C_19_H_30_Cl_2_CrN_5_O_9_], a peak at *m/z* = 576.049 (found = 576.070), and a base peak at *m/z* = 558.039 (found = 558.060) were associated with [C_19_H_28_Cl_2_CrN_5_O_8_] and [C_19_H_26_Cl_2_CrN_5_O_7_] fragments, respectively. Two base peaks at *m/z* = 255.065 (found = 253.050) and *m/z =* 130.109 (found = 129.100) were associated with the chrysin (C_15_H_9_O_4_) and metformin (C_4_H_911_N_5_) ligands, respectively, confirming the coordination of the utilized ligands according to the designed complex (Scheme 1). Moreover, the presence of *m*+4 type peaks to the *m/z* peaks confirmed the coordination of two chlorides to the Cr(III) center (Supplementary Figures S6, S7 of the Supporting Material).

### 3.6 X-ray diffraction analysis

The XRD patterns of **Cr1** and **Cr2** mixed ligand complexes showed diffraction peaks in the range 2θ = 5°–80°, indicating the polycrystalline phases of the synthesized complexes (Supplementary Figure S9). The **Cr1** complex was an orthorhombic crystal system with spacing group P b c a and lattice parameters 16.2680 Å, 22.3369 Å, 24.0479 Å, 90°, 90°, and 90° for a, b, c, α, β, and γ, respectively. In contrast, **Cr2** followed a monoclinic crystal system and P 1 21/c 1 space group with lattice parameters 7.3135 Å, 7.6312 Å, 20.5697, Å, 90°, 99.462°, and 90°, respectively for a, b, c, α, β, and γ.

The average crystallite sizes of the complexes were calculated according to the Debye–Scherrer equation (Holzwarth and Gibson, 2011). The average crystalline sizes of the complexes were 21.453 and 19.600 nm, respectively, for **Cr1** and **Cr2**. The percent crystalline index of the complexes was calculated using Equation (2).

$$Crystalline\;Index\;\left(CI\right)=\frac{Ac}{Ac+Aa}\times 100\%,$$ (2)

where Ac is the area of crystalline and Aa is the area of the amorphous part of the materials.

Accordingly, the synthesized Cr(III) mixed ligand complexes showed 66.51% and 31.37% crystallinity for **Cr1** and **Cr2,** respectively. The numbers of dislocation lines per unit area of the crystal and dislocation density (δ∗) of the complexes were calculated from their relation to the average crystalline size (D) of the complexes (El-Sonbati et al., 2019; El-Sonbati et al., 2021) using Equation (3).

$$Dislocation\;density\;\left(\delta *\right)=\frac{1}{D^{2}}.$$ (3)

The calculated dislocation density values were 2.324 × 10^−3^ and 2.603 × 10^−3^ nm^-2^ for **Cr1** and **Cr2** complexes, respectively, representing a crystalline material with relatively less dislocation density and less irregularity within the structure. Previous research work by El-Sonbati et al. (2021) reported dislocation densities ranging from 3.00 × 10^−4^–2.10 × 10^−3^ nm^-2^ for Cu(II), Ni(II), Mn(II), and UO_2_II) mixed ligand polycrystalline complexes.

### 3.7 Thermogravimetric analysis

**FIGURE 3:**
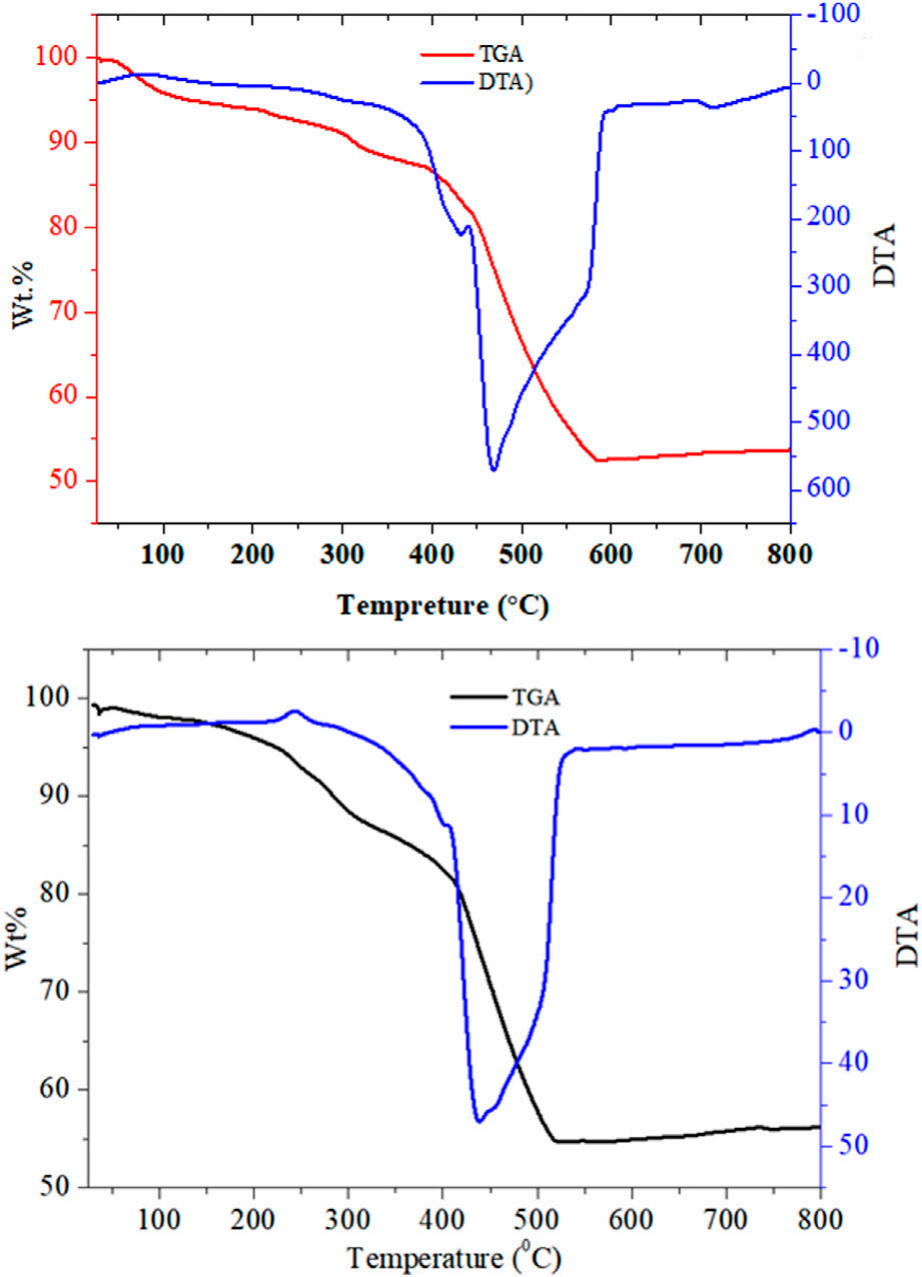
TGA/DTA graphical presentations of the **Cr1** (top) and **Cr2** (bottom) complexes.

The thermal stabilities of the **Cr1** and **Cr2** complexes were identified based on the thermogravimetric curves in the temperature range between 25°C and 800°C (Table 2; Figure 3). The **Cr1** complex thermally decomposed into two main degradation steps: *i)* from 51.2°C to 213.2°C (DTG_max_ = 126°C), the weight loss was 7.21% (calcd. 7.20%), corresponding to the release of water molecules, *ii)* from 295.2°C to 590.4°C (DTG_max_ = 465°C) due to C_15_H_8_O_3_ + 2Cl groups with a total weight loss of 41.63% (calcd. 41.60%) (Ndong et al., 2020).

**TABLE 2:** Temperature range values for decomposition and corresponding weight loss values.

	Decomposition temp. (^°^C)	Mass loss (%)		DTG_max_ (^o^C)	Interpretation
		Obsd	Calcd		
**Cr1**	51–213	7.21	7.19	126	Loss due to water molecules
	295–590	41.63	41.60	465	Loss due to C_15_H_8_O_3_ + 2Cl groups
**Cr2**	55–267	9.11	9.80	243	Loss due to water molecules
	275–411	11.76	11.74	400	Release of the chlorine moiety
	420–524	24.42	24.40	441	Decomposition of C_4_H_11_N_5_ + OH^−^ organic moiety

The **Cr2** complex thermally decomposed into three main degradation steps: *i)* from 55.56°C to 267.41°C (DTG_max_ = 243°C), the weight loss was 9.11% (calcd. 9.10%), corresponding to the release of water molecules; *ii)* from 275.57°C to 411.07°C (DTG_max_ = 400°C), associated with the decomposition of the two chlorine moiety, with a total weight loss of 11.76% (calcd. 11.73%); *iii)* from 420°C to 524°C (DTG_max_ = 441°C) and a weight loss of 24.42% (calcd 24.40%), corresponding to the decomposition of C_4_H_11_N_5_ + OH organic moiety with the final residue of chromium oxide (Ndong et al., 2020).

### 3.8 SEM-EDX analysis

**FIGURE 4:**
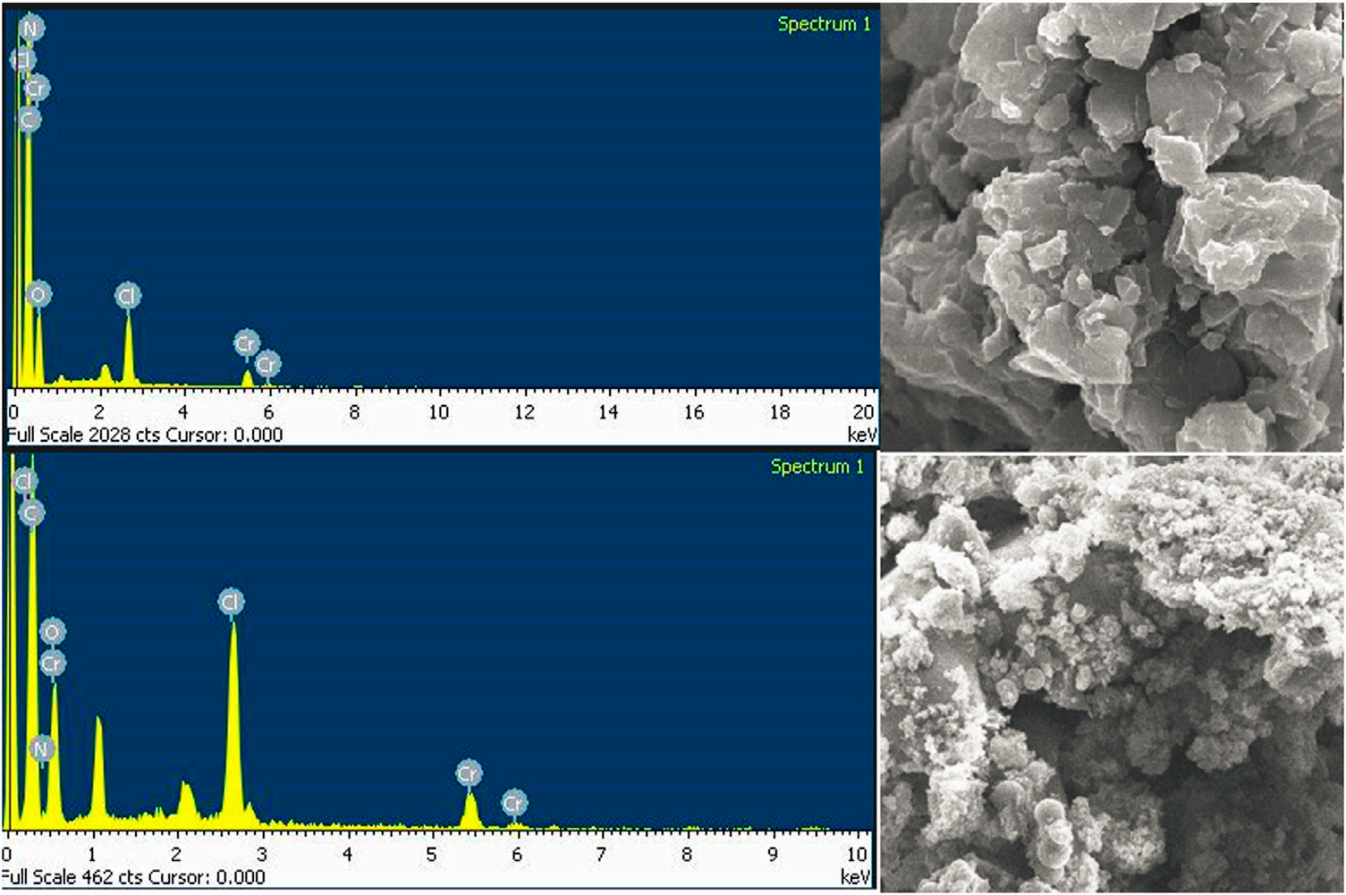
SEM-EDX images of the **Cr1** (top) and **Cr2** (bottom) complexes.

The elemental compositions of the reported complexes (**Cr1** and **Cr2**) were obtained from energy-dispersive X-ray (EDX) analysis. The SEM image of the synthesized complexes showed agglomerated particles and the presence of non-uniform-sized small grains. The EDX spectra of **Cr1** and **Cr2** showed characteristic signals for carbon, nitrogen, oxygen, chlorine, and chromium, which were associated with the atoms present in the ligands and the metal center, confirming the presence of the complexes waving the CHCrNOCl chemical composition of the complexes (Figure 4).

### 3.9 Quantum chemical analysis

Quantum chemical descriptors vital for correlating the energy, structure, and reactivity characteristics of the synthesized Cr(III) complexes were calculated from the frontier molecular orbitals (FMOs) HOMO, and LUMO (Table 3. The reactivities of the ligands and the synthesized complexes were determined using the highest occupied molecular orbital (HOMO) and lowest unoccupied molecular orbital (LUMO) band gap energy. The HOMO and LUMO eigenvalues and band gap energy are strongly associated with biological activities such as antibacterial, cytotoxicity, and antioxidant activities (Alem et al., 2022; Damena et al., 2023). The band gap energy (eV) of the ligands and their metal complexes were 5.988, 4.403, 2.609, and 3.452 eV, respectively, for metformin (Met), chrysin (Cry), **Cr1**, and **Cr2**. The metal complexes showed decreased band gap energy supported by increased global softness (Table 3), inferring the biological significance of the synthesized metal complexes relative to the ligands alone. The higher eigenvalue of the HOMO (−5.959 eV) and high dipole moment (10.838 Debye) of **Cr2** were major contributors to its better *in vitro* antibacterial activity (*vide supra*).

**TABLE 3:** Quantum chemical descriptors of the ligands and Cr(III) complexes: band gap (eV), electronegativity (eV), electronic chemical potential (eV), global chemical hardness (eV), global softness (*σ* = eV^-1^), global electrophilicity index (eV), nucleophilicity index (eV^-1^), and dipole moment (Debye).

Cpd	HOMO	LUMO	*E_g_*	*χ*	*μ*	*Η*	*Σ*	*Ω*	*Nu*	Dipole moment
Met	−6.440	−0.452	5.988	3.446	−3.446	2.994	0.167	1.983	0.504	5.752
Phen	−6.690	−1.935	4.755	4.313	−4.313	2.377	0.210	3.912	0.256	5.192
Cry	−6.555	−2.152	4.403	4.353	−4.353	2.202	0.227	4.304	0.232	7.884
**Cr1**	−4.989	−2.380	2.609	3.684	−3.684	1.304	0.383	5.203	0.192	0.062
**Cr2**	−5.959	−2.508	3.451	4.234	−4.234	1.725	0.290	5.194	0.193	10.838

Met, metformin; Phen, 1,10-phenanthroline; Cry, chrysin.

According to the hard–soft–acid–base (HSAB) theory, hard acids prefer to coordinate with hard bases and soft acids with soft bases (Ismael et al., 2020; Alem et al., 2022). Hence, DNA, enzymes, and proteins are soft biological molecules targeted for drug discovery. The small band gap energy together with large softness owing to the synthesized metal complexes make them promising therapeutic agents.

Chemical potential (*μ*) is the tendency of an electron to leave a stable system. A negative chemical potential indicates that a stable complex that does not spontaneously decompose into its constituent element is stable, whereas hardness (*η*) measures the resistance to changes in the distribution of electrons within a molecule. It is characterized by compounds with large HOMO–LUMO gaps possessing less reactivity (Choudhary et al., 2019). In this study, metal complexes showed negative chemical potentials (*μ*) of −3.684 and −4.234 eV, respectively, for **Cr1** and **Cr2**, inferring that **Cr2** followed a stable system.

### 3.10 Frontier molecular orbital (FMO) analysis

**FIGURE 5:**
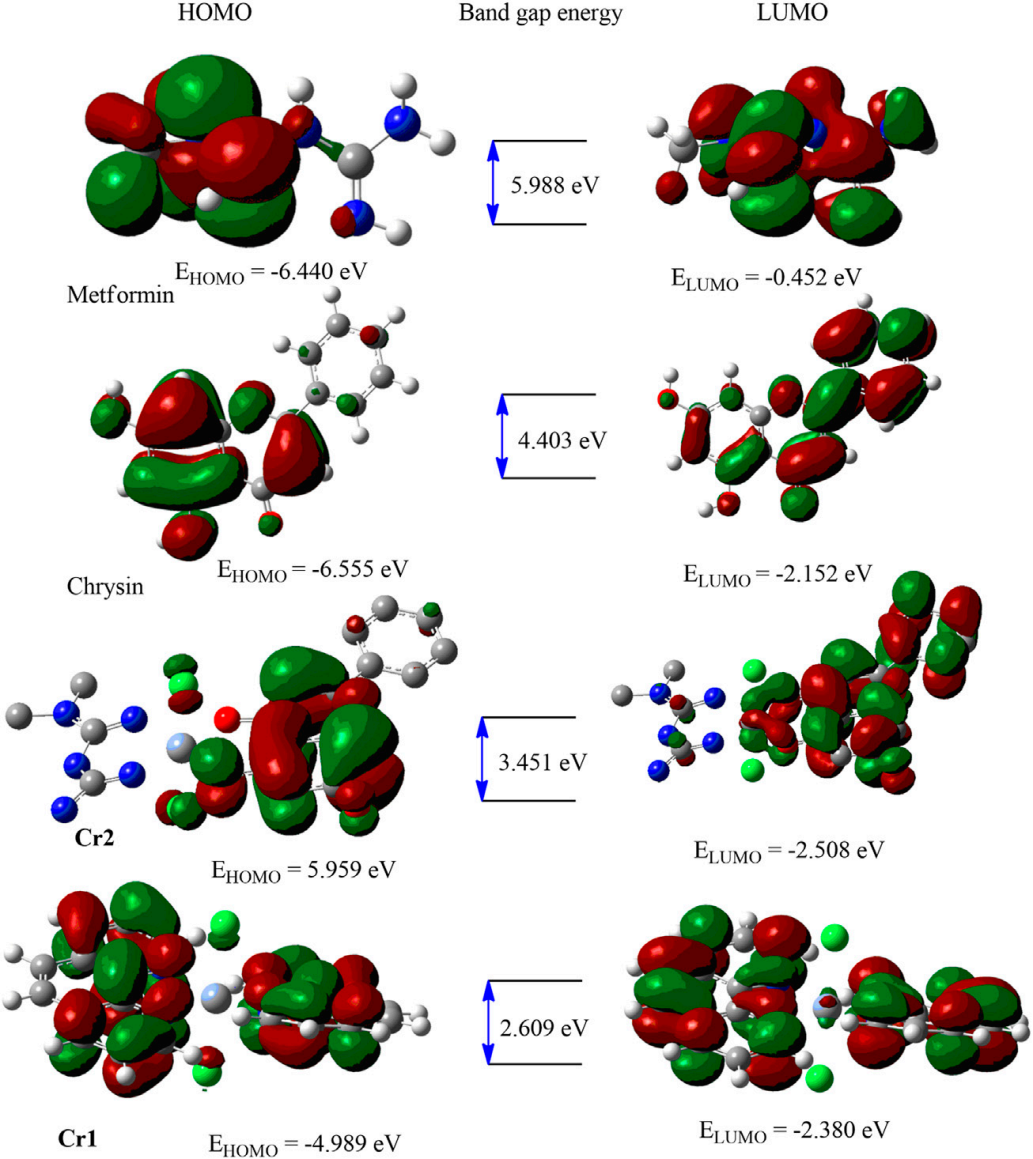
HOMO–LUMO distributions of the ligands and metal complexes.

The B3LYP-calculated HOMO–LUMO distributions of the ligands and the corresponding metal complexes (**Cr1** and **Cr2**) are presented in Figure 5 and Supplementary Figure S8. In **Cr1**, the uniform distribution of the HOMO–LUMO confirmed the presence of ligand–ligand charge transfer *via* the Cr(III) metal center. This dominant charge transfer masked the expected d→d electronic transition. In the **Cr2** complex, the HOMO–LUMO isodensity showed the concentration of the HOMO on chrysin and LUMO on the chrysin and metal center, inferring the possible intra-ligand and ligand-to-metal charge transfer.

### 3.11 Biological activity evaluation

#### 3.11.1 Cytotoxicity evaluation against MCF-7 cells

**FIGURE 6:**
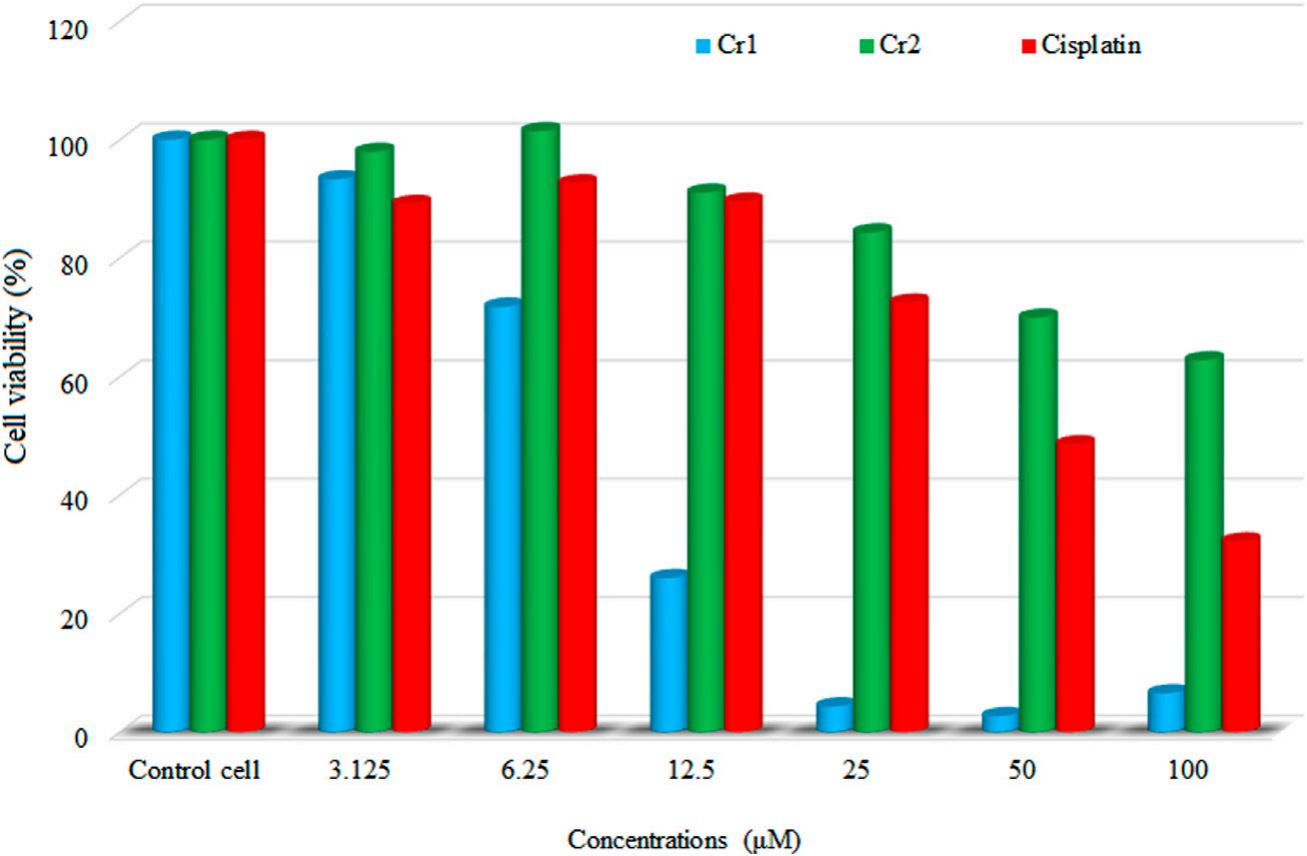
Cell viability profile of the synthesized complexes against MCF-7 cells.

Cr(III) complexes have been used as efficient cancer therapeutic agents both *in vivo* and *in vitro via* the reactive oxygen species (ROS) generation mechanism (Chen et al., 2021). In contrast, flavonoid (Renfrew, 2014; Kasprzak et al., 2015) and 1,10-phenanthroline-based metal complexes cause cytotoxicity in cancer cells (Bravo-Gómez et al., 2009; Ruiz-Azuara and Bravo-Gomez, 2010; Buchtík et al., 2012; Reina et al., 2021). A low incidence of cancer has been reported among patients with diabetes treated with metformin (Abdelrahman et al., 2021), suggesting that metformin reduces cancer risk. Some of the proposed mechanisms of anticancer activity of metformin are based on its ability to form an adduct with glutathione and interactions with mitochondrial copper ions (Abdelrahman et al., 2021). The viability of the MCF-7 cell line was evaluated by MTT assay with six concentrations of test compounds in triplicate (Figure 6 and Supplementary Table S1).

**FIGURE 7:**
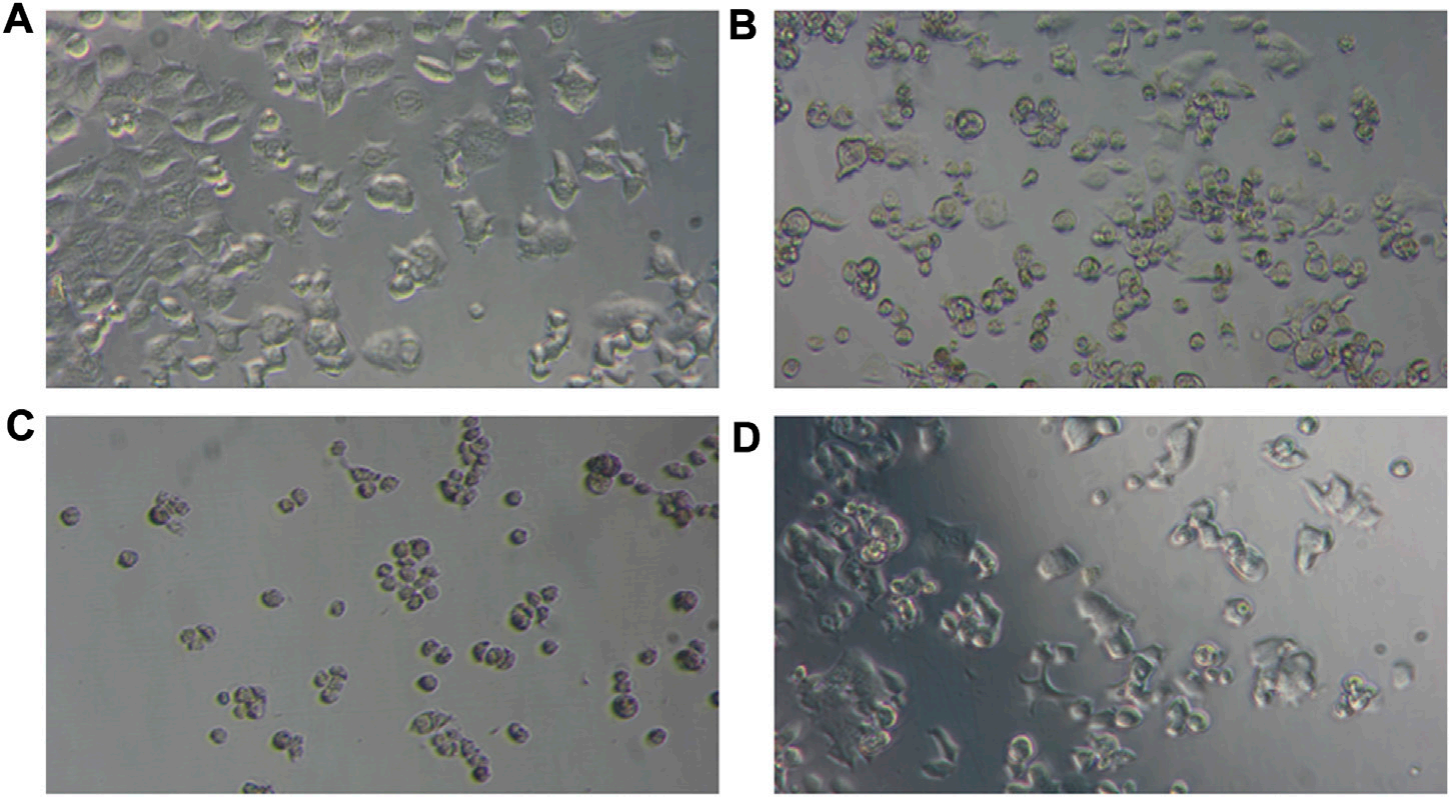
Photomicrographs of the cellular morphology of **(A)** untreated MCF-7 and the changes induced by **(B)** cisplatin, **(C) Cr1**, and **(D) Cr2** using 50 μM.

In this study, a natural flavonoid, chrysin, and metformin coordinated Cr(III) complexes were synthesized and subjected to cytotoxicity evaluation against the human breast adenocarcinoma cancer cell line (MCF-7) at a range of concentrations (3.125–100 µM). Although both complexes showed promising cytotoxicity results, the **Cr1** complex showed better cytotoxic activity, with an IC_50_ value of 8.08 μM compared to **Cr2** (IC_50_ = 30.85 μM) and cisplatin (IC_50_ = 18.62 μM). A lower cell viability indicates a higher cytotoxic potential of the test compound (Ramasamy et al., 2022); i.e., a test compound is considered cytotoxic for vitality percentage <70% and non-cytotoxic for values >70% (Cannella et al., 2019; Ismael et al., 2020; Ramasamy et al., 2022). Therefore, the reported Cr(III)-based compounds in this study are considered cytotoxic with IC_50_ values in the range of those previously reported (Bravo-Gómez et al., 2009; Alem et al., 2022). Comparing the ligand composition of the complexes, **Cr1** with 1,10-phenanthroline showed enhanced bioactivity consistent with the previously reported 1,10-phenanthroline-based cytotoxic metal complexes (Bravo-Gómez et al., 2009; Buchtík et al., 2012; Nunes et al., 2020; Reina et al., 2021). The cellular morphology of the microscopic images of the MCF-7 cells (untreated) and treated with 50 μM **Cr1**, **Cr2**, and cisplatin for 24 h is provided in Figures 7A–D.

MCF-7 cells are characterized by their epithelial-like cell morphology with irregular and polygonal shapes (Andiappan et al., 2018; Razak et al., 2019). This morphology was observed in untreated MCF-Cells (Figure 7A). However, after treatment with 50 μM **Cr1**, **Cr2**, and cisplatin for 24 h, the morphology changed, with the cells appearing round to oval in shape, while the cell density was also reduced. The effect was more pronounced for the **Cr1** complex even at lower concentrations (12.5 μM). Generally, the microscopic and cell viability results obtained from this work demonstrated that the reported metal complexes are cytotoxic, as reported for other metal complexes (Andiappan et al., 2018; Razak et al., 2019; Nunes et al., 2020; Alem et al., 2022).

#### 3.11.2 Molecular docking analysis against estrogen receptor alpha

**FIGURE 8:**
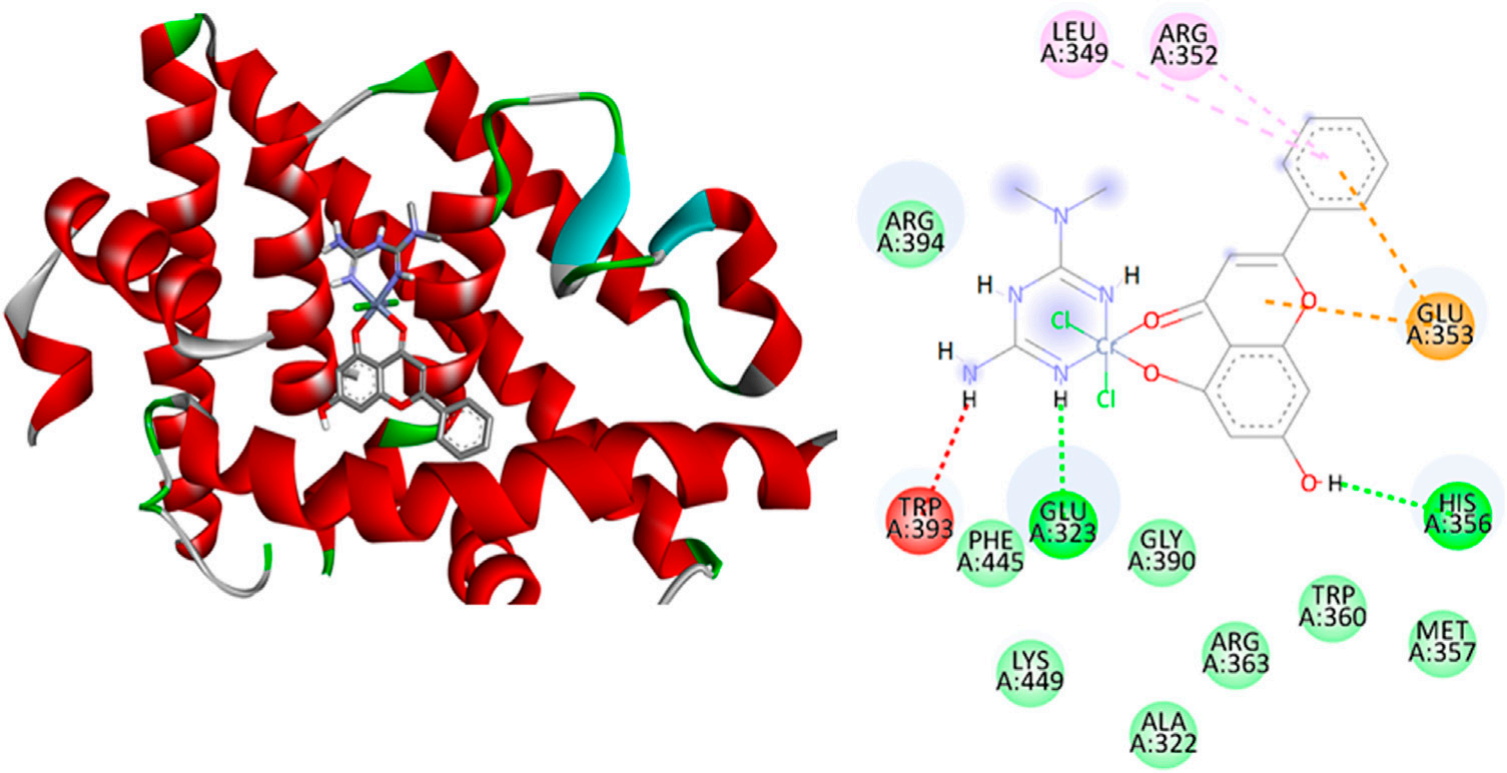
Binding interactions of **Cr2** against estrogen receptor alpha (ERα; PDB: 5G4).

The 2D and 3D interaction of the synthesized complexes (**Cr1** and **Cr2**) with estrogen receptor alpha (ERα; PDB:5G4) residual amino acids together with a binding affinity (kcal/mol) and the inhibition constant (Ki) parameters are given in Table 4; Figure 8 and Supplementary Figure S10 of the Supporting Material. **Cr1** demonstrated a binding energy and an inhibition constant of −6.83 kcal/mol and 9.93 µM, respectively, with a total of 14 van der Waals (Phe 445, Gly 390, Ile 386, Leu 387, Trp 360, and Asn 359) and π–alkyl/π–ion (Met 357, His 356, Arg 363, Lys 449, Arg 394, Glu 353, Glu 323, and His 356) interactions (Figure 8). **Cr2** showed two hydrogen bonding (Glu 323 and His 356), 16 van der Waals (Arg 394, Phe 445, Lys 449, Gly 390, Ala 322, Arg 263, and Met 357), and π–alkyl/π–ion (Arg 394, Lee 349, Arg 352, Glu 353, Glu 353, Glu 323, Glu 323, Trp 393, and Trp 393) interactions. Arg 394, Glu 353, Leu 387, and Leu 391 are the most important active site amino acids of ERα that participate in hydrogen bonding and π–alkyl interactions (Pang et al., 2018). The synthesized complexes showed interaction with three of the amino acids (Arg 394, Glu 353, and Leu 387), with high interactions for **Cr1**. The obtained residual amino acid interactions showed that the number of interactions affected the binding affinity and *K*i. The results further showed that the **Cr1** > Cisplatin > **Cr2,** in line with the cytotoxicity activity of the complexes.

**TABLE 4:** Molecular docking scores and the corresponding prominent residual amino acid interactions of the complexes against estrogen receptor alpha (ERα; PDB: 5G4).

Cpds	Rmsd	Binding energy (kcal/mol)	Inhibition constant (*K*_i_) (µM)	H-bonding	van der Waals	π–alkyl/π–ion
**Cr1**	1.14	−6.83	9.93	-	Phe 445, Gly 390, Ile 386, Leu 387, Trp 360, and Asn 359	Met 357, His 356, Arg 363, Lys 449, Arg 394, and Glu 323
**Cr2**	1.35	−6.26	22.99	-	Phe 445, Gly 390, Ile 386, Leu 387, Trp 360, and Asn 359	Arg 390, Lys 449, Glu 357, Arg 363, His 356, and Met 357
**Cisplatin**	0.39	−6.32	23.42	Ser 468 and Asp 374	Lys 467	Thr 371 and Glu 471

### 3.12 Antimicrobial activity studies

**FIGURE 9:**
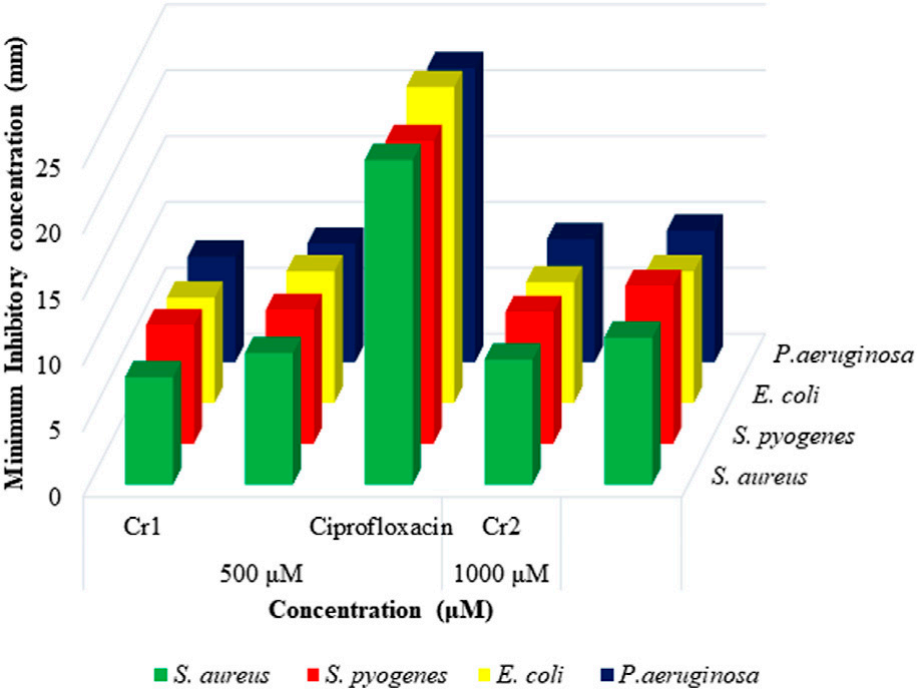
Antibacterial activity evaluation graph for **Cr1** and **Cr2** complexes.

All synthesized complexes showed minimum inhibition zones ranging from 8.00 to 12.00 mm, inferring that the complexes were active against the tested bacterial strains (Supplementary Table S2), as cultured bacteria with halos >7 mm were considered susceptible to the tested complex (Alam et al., 2010; Allouche, 2012; Omolo et al., 2014). However, a high difference between the MIZ of the complexes and the control, ciprofloxacin, was observed (Figure 9).

#### 3.12.1 Molecular docking analysis against *Staphylococcus aureus* and *Escherichia coli*

**FIGURE 10:**
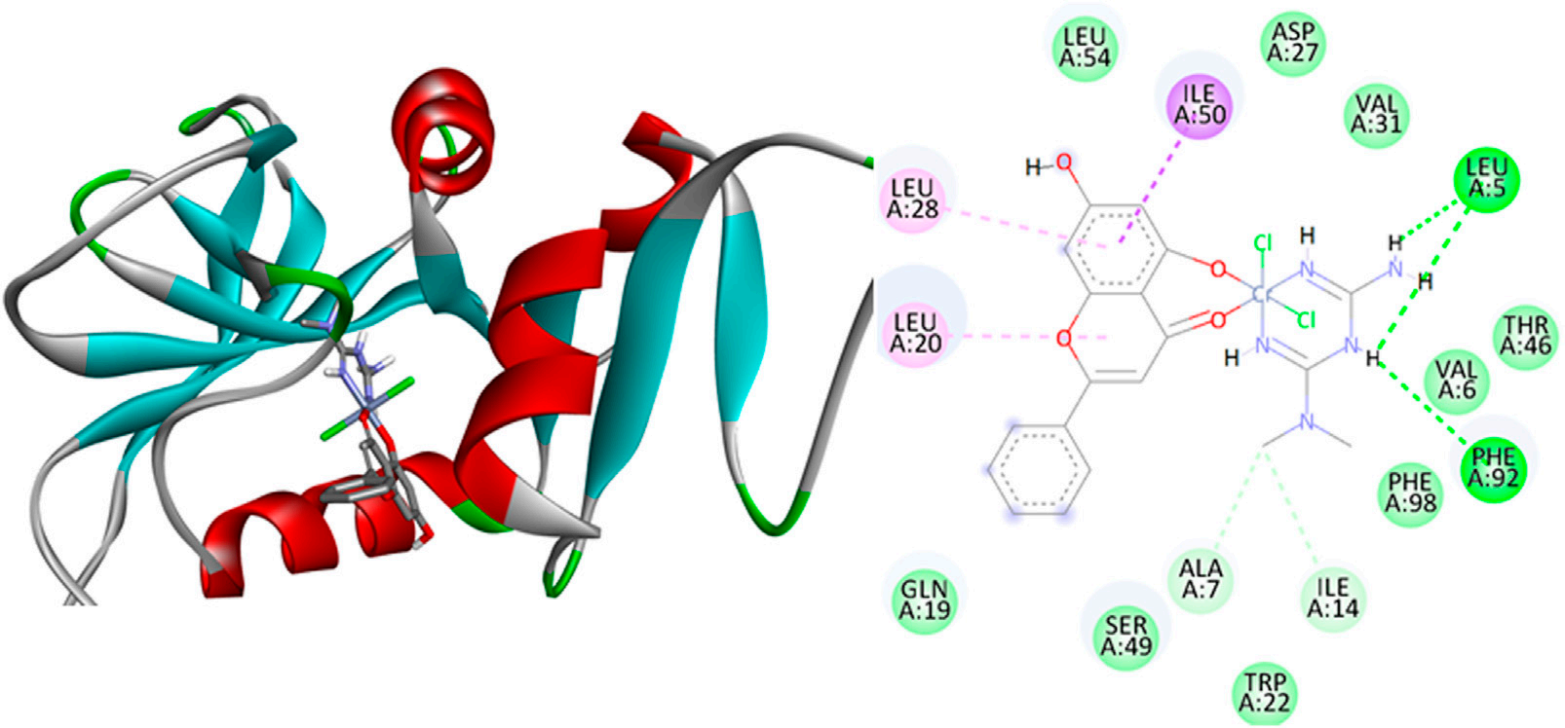
Binding interactions of **Cr2** against *Staphylococcus aureus* (PDB: 2w9h).

Molecular docking studies fundamentally define the binding modes of ligand interaction at the active site of the protein (Kouser et al., 2022). In this study, the synthesized Cr(III)-centered mixed ligand complexes were subjected to molecular docking studies against bacterial strains on which the complexes showed better *in vitro* antibacterial activity. The binding interactions with the complexes showed that **Cr2** created hydrogen bonding interactions with Leu 5 and Phe 92 amino acid residues (Supplementary Tables S2, S4 and Figure 10). Moreover, it created 14 interactions, nine of which were van de Waals (Leu 54, Asp 27, Val 31, Thr 46, Val 6, Phe 98, Trp 22, Ser 49, and Gln 19) and five of which were π–alkyl/π–ion (Leu 28, Leu 20, Ala 7, Ile 4, and Ile 50) with a binding energy and inhibition constant of −8.82 kcal/mol and 0.30 µM, respectively. The presence of a greater number of interactions, high binding energy, and small inhibition constant of **Cr2** against *S. aureus* make this complex show better *in vitro* antibacterial activity compared to **Cr1** (Supplementary Table S3 and Supplementary Figure S11). Similarly, the docking study of **Cr2** against *E. coli* protein showed high binding energy (−7.51 kcal/mol), minimum inhibition constant (2.78 µM), two hydrogen bonds (Gly 77 and Asn 46), eight van der Waals interactions (Val 44, Thr 165, Gly 75, Glu 50, Pro 79, Asp 49, Ile 94, and Met 166), and seven π–alkyl/π–ion interactions (Gly 77, Val 167, Val 43, Ala 47, Asp 73, Val 71, and Asn 46 amino acid residues) (Supplementary Table S4 and Figure 10, Supplementary Figure S11–S14). The obtained results in all types of bacterial strains are consistent with the minimum zone of inhibition obtained in the *in vitro* evaluations of the antibacterial activity.

#### 3.12.2 DPPH radical scavenging activity analysis

**FIGURE 11:**
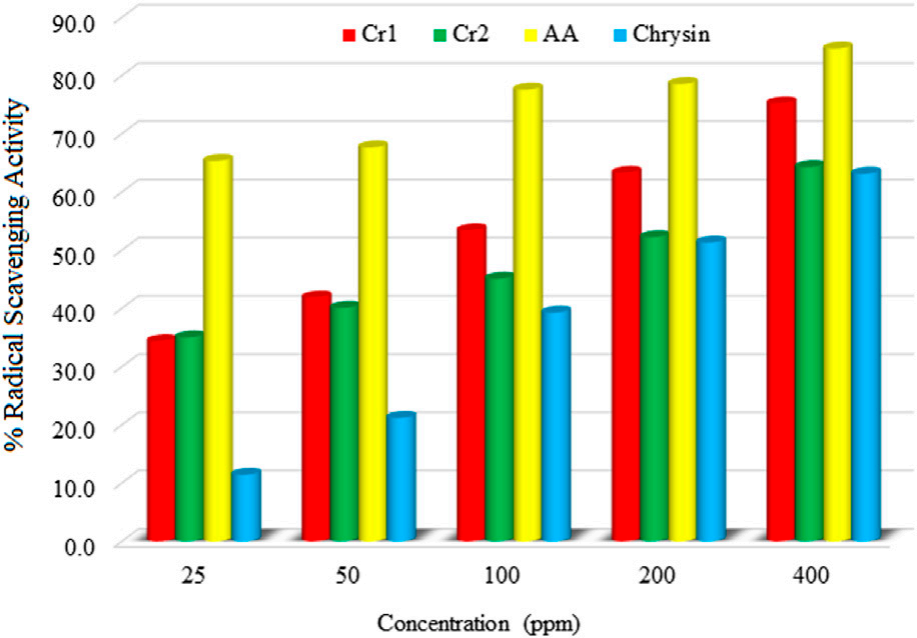
Antioxidant profiles of the synthesized metal complexes (**Cr1** and **Cr2**).

The graphical presentation of the free radical scavenging activity of the free ligand chrysin and the synthesized complexes (**Cr1** and **Cr2**) were evaluated using a DPPH assay (Figure 11. Higher antioxidant activities (34.370%–75.179% DPPH free radical scavenging) of the metal complexes than the free ligand (11.398%–63.118%) were observed. The extended conjugation because of the metal coordination to the ligands might have enhanced the proton transfer process observed in the increased antioxidant activity of the metal complexes over the free ligand chrysin. The antioxidant activity against DPPH free radical scavenging followed the order AA > **Cr1** > **Cr2** > chrysin with percent radical scavenging activities of 84.624, 75.179, 64.245, and 63.118% for AA, **Cr1**, **Cr2**, and chrysin, respectively, at 400 ppm.

## 4 Conclusion

Organic–inorganic hybrid salts of Cr(III) complexes containing the natural flavonoid chrysin were synthesized. Spectroscopic (FTIR and UV/Vis), microscopic (SEM-EDX), thermogravimetric (TGA/DTA), mass spectrometric, pXRD, and molar conductance measurements were used to characterize the synthesized complexes. The coordinations of the ligands to the metal ions were confirmed using FTIR and UV/Vis spectral data. A molar conductance study was used to confirm the ionic/non-ionic nature of the complexes. The molecular weights of the metal complexes were confirmed by mass spectrometry. SEM-EDX was used to confirm the composition of the elements in the synthesized metal complexes. The pXRD patterns of the complexes in their powder showed polycrystalline natures with crystalline average sizes of 21.453 and 19.600 nm, for **Cr1** and **Cr2**, respectively. Biologically, **Cr1** showed significant cytotoxicity against the MCF-7 cell line, with an IC_50_ value of 8.08 μM compared to **Cr2** (IC_50_ = 30.85 μM) and cisplatin (IC_50_ = 18.62 μM). The overall results showed that the Cr(III) complexes reported in this study are promising cytotoxic drug candidates. However, further *in vivo* cytotoxicity studies against the MCF-7 cell line and toxicity studies are recommended.

## Data Availability

The datasets presented in this study can be found in online repositories. The names of the repository/repositories and accession number(s) can be found in the article/Supplementary Material.
