# Supplementary material for: Organic–inorganic hybrid salt and mixed ligand Cr(III) complexes containing the natural flavonoid chrysin: Synthesis, characterization, computational, and biological studies
Source: Front Chem. 2023 Apr 12;11:1173604. doi: 10.3389/fchem.2023.1173604 (PMC10130586; doi:10.3389/fchem.2023.1173604)
Supplement: Supplementary file 1 [file DataSheet1.PDF]

Organic-Inorganic Hybrid Salt and Mixed Ligand Cr(III) Complexes Containing the Natural Flavonoid Chrysin: Synthesis, Characterization, Computational and Biological Studies

Mamaru Bitew Alem,[1,]* Tegene Desalegn,[1,]* Tadewos Damena,[2] Enyew Alemayehu Bayle,[3,4] Moses O. Koobotse,[5] Kennedy J. Ngwira,[6] Japheth O. Ombito,[7] Matshediso Zachariah,[5] and Taye B. Demissie[7,]*

[1]Department of Applied Chemistry, Adama Science and Technology University, P.O.Box 1888, Adama, Ethiopia

[2]Department of Chemistry, Wachemo University, P.O.Box 667, Hossana, Ethiopia

[3]Graduate Institute of Applied Science and Technology, National Taiwan University of Science and Technology, 10607 Taipei, Taiwan

[4]Department of Chemistry, Debre Markos University, P.O.Box 269, Debre Markos, Ethiopia

[5]School of Allied Health Professions, University of Botswana, P/Bag UB 0022, Gaborone, Botswana

[6]Molecular Sciences Institute, School of Chemistry, University of the Witwatersrand, PO Wits, 2050, Johannesburg, South Africa

[7]Department of Chemistry, University of Botswana, P/Bag UB 0022, Gaborone, Botswana

Correspondence: mamaru2005@gmail.com (MBA)
ORCID Id: https://orcid.org/0000-0002-1880-8572
tegened@yahoo.com (TD)
ORCID Id: https://orcid.org/0000-0003-0239-8326
demissiet@ub.ac.bw (TBD)
ORCID Id: https://orcid.org/:0000-0001-8735-4933

Figures **page**

Figure S1. FTIR Spectra of the ligands (1,10-phen, Cry) and their metal complexes (**Cr1**) 3
Figure S2. FTIR specra of metformin, chrysin and **Cr2** complex 4
Figure S3. UV-Vis and TD-DFT calculated absorption spectrum of the ligands (chrysin and 1,10-phenanthroline). 5
Figure S4. UV-Vis and TD-DFT calculated absorption spectrum of Metformin 6
Figure S5. UV-Vis and TD-DFT calculated absorption spectrum for **Cr2** alternative structure ... 7
Figure S6. Mass spectrometric spectra of complex **Cr1**.. 8
Figure S7. Mass spectrometric spectra of complex **Cr2.**... 9
Figure S8. HOMO-LUMO distribution and band gap energy of 1,10-phenathroline 10
Figure S9. Powder x-ray diffraction patterns for **Cr1** and **Cr2** mixed ligand complexes 11
Figure S10. 3D and 2D representation of the interaction of **Cr1** in the binding pocket amino acid residue of ERa (ERα; PDB: 5GS4) 12
Figure S11. The binding interactions of **Cr1** against E. coli (PDB: 6F86) 14
Figure S12. The binding interactions of **Cr1** against S. aureus (PDB: 2w9h) 15
Figure S13. The binding interactions of **Cr2** against E. coli (PDB: 6F86) 16
Figure S14. The binding interactions of **Ciprofloxacin** against E. coli (PDB: 6F86) 17

Tables **Page**

Table S1. Cytotoxicity measurements for percent cell viability of MCF-7 cell line 10
Table S2. Antibacterial activity data of **Cr1** and **Cr2** complexes (MIZ (mm) ± SD) 10
Table S3. Molecular docking scores and the corresponding prominent residual amino acid interactions of the complexes against *S. aureus* (PDB: 2w9h) 13
Table S4. Molecular docking scores and the corresponding prominent residual amino acid interactions of the complexes against *E. coli* (PDB: 6f86) 13
Table S5. B3LYP optimized geometry of Cr1 18
Table S6. B3LYP Optimized geometry of Cr2 19

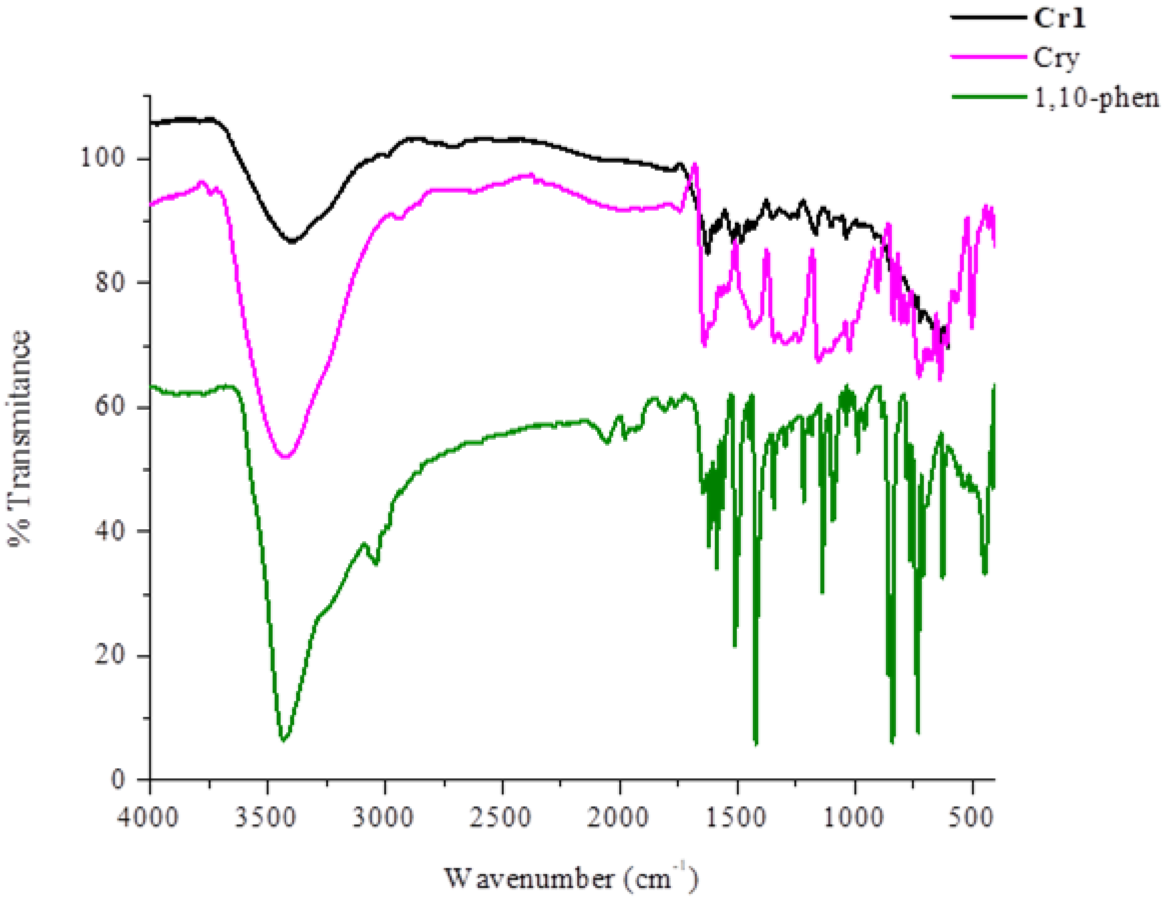

Figure S1. FTIR Spectra of the ligands (1,10-phen, Cry) and their metal complexes (**Cr1**)

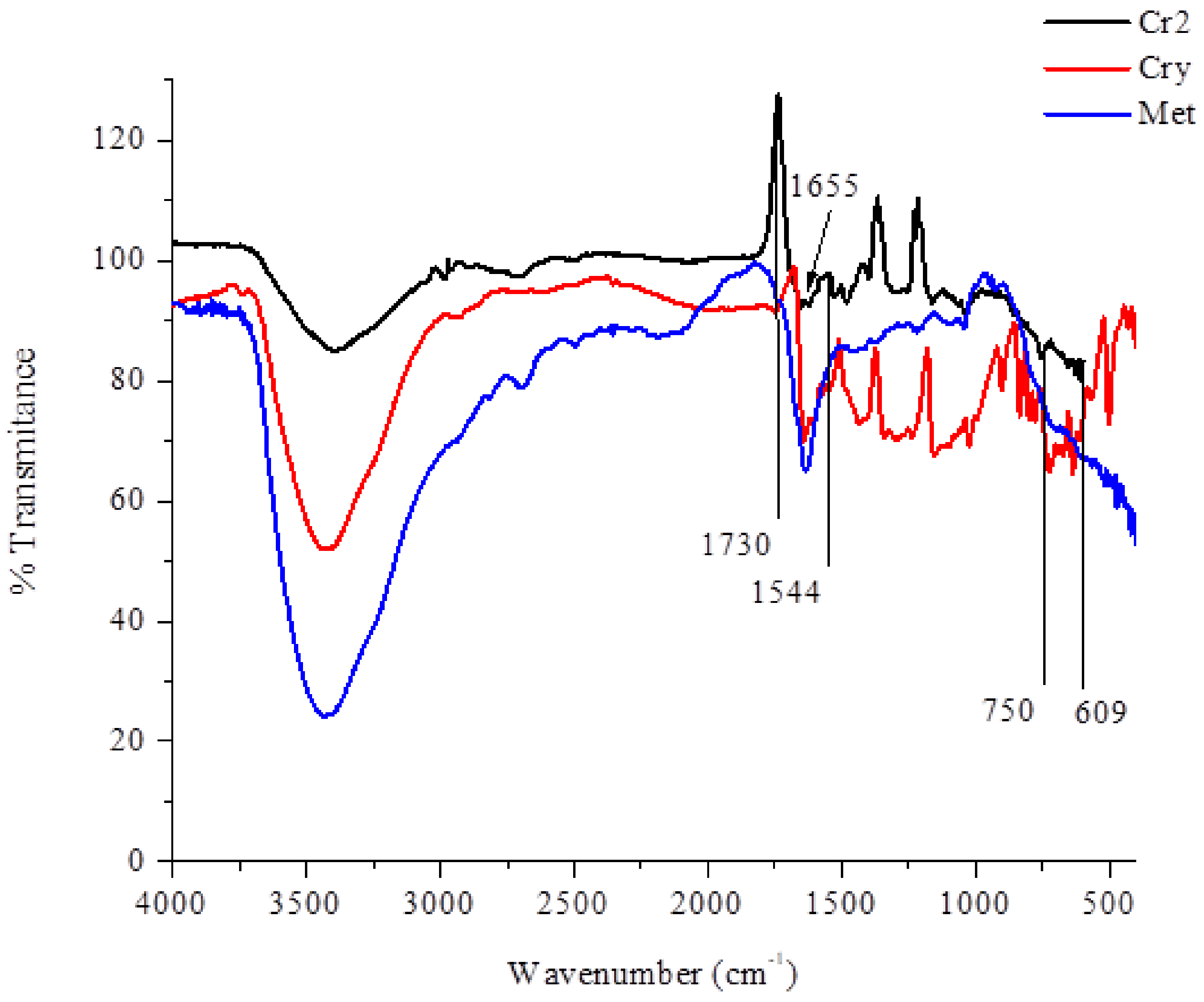

Figure S2. FTIR specra of metformin, chrysin and **Cr2** complex

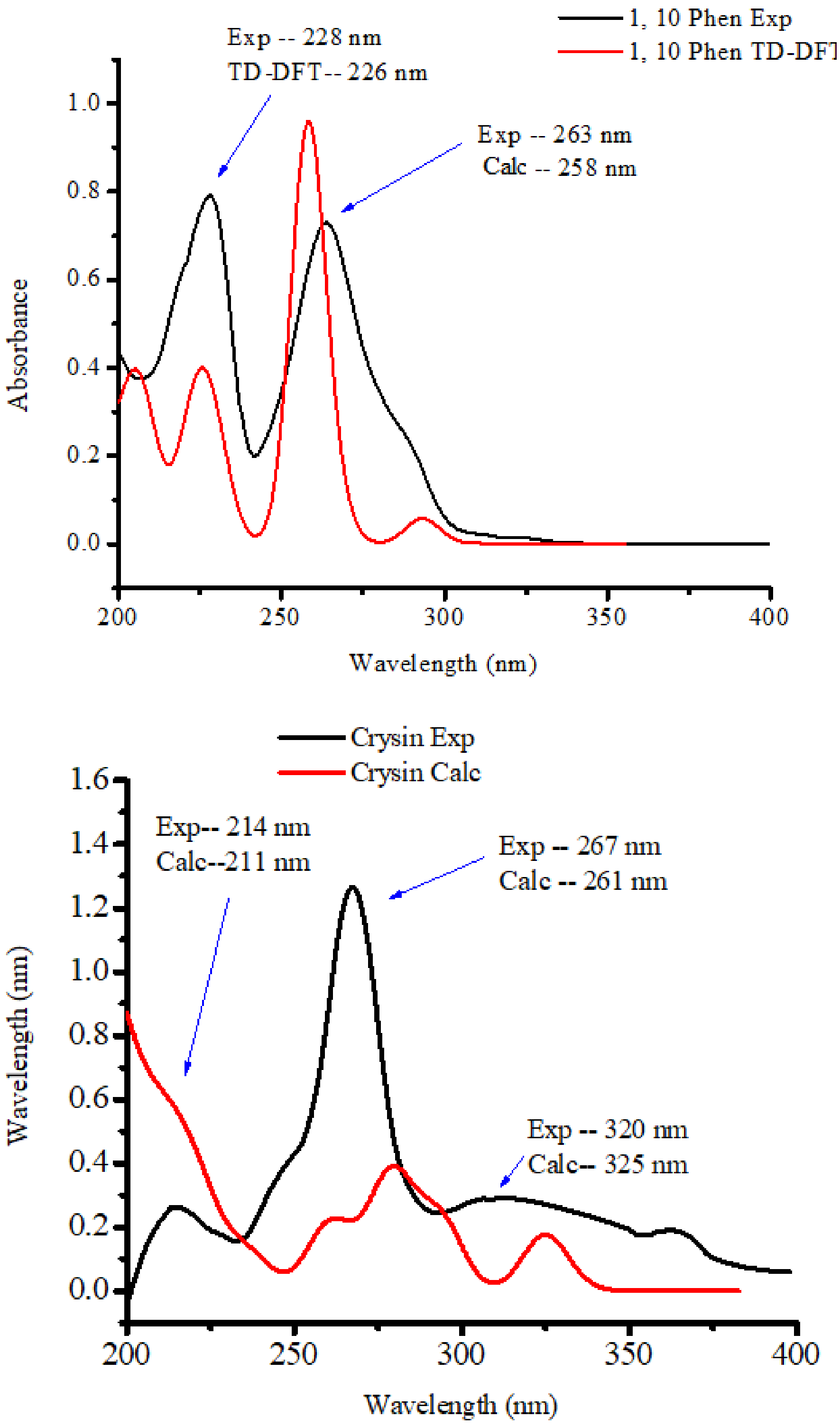

Figure S3. UV-Vis and TD-DFT calculated absorption spectrum of the ligands (chrysin and 1,10-phenanthroline).

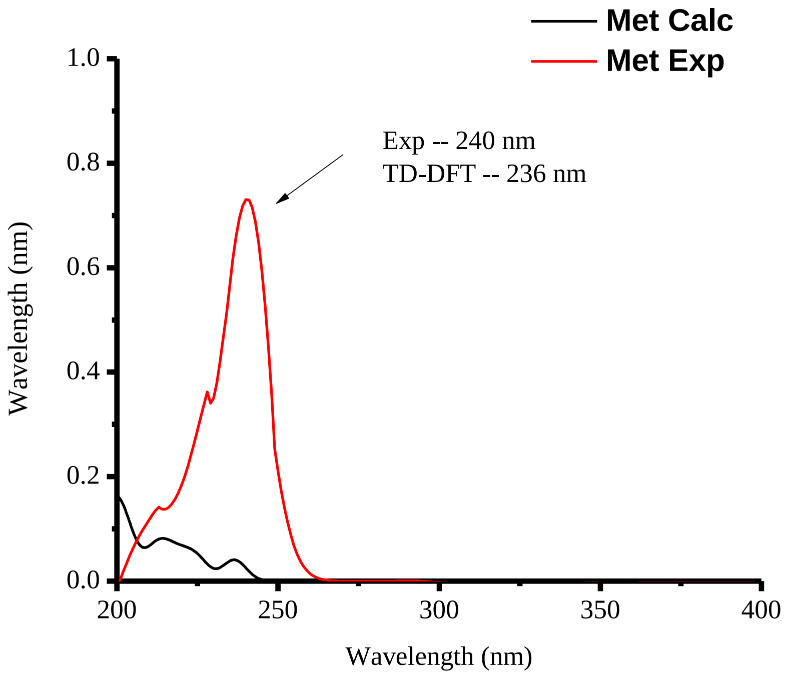

Figure S4. UV-Vis and TD-DFT calculated absorption spectrum of Metformin

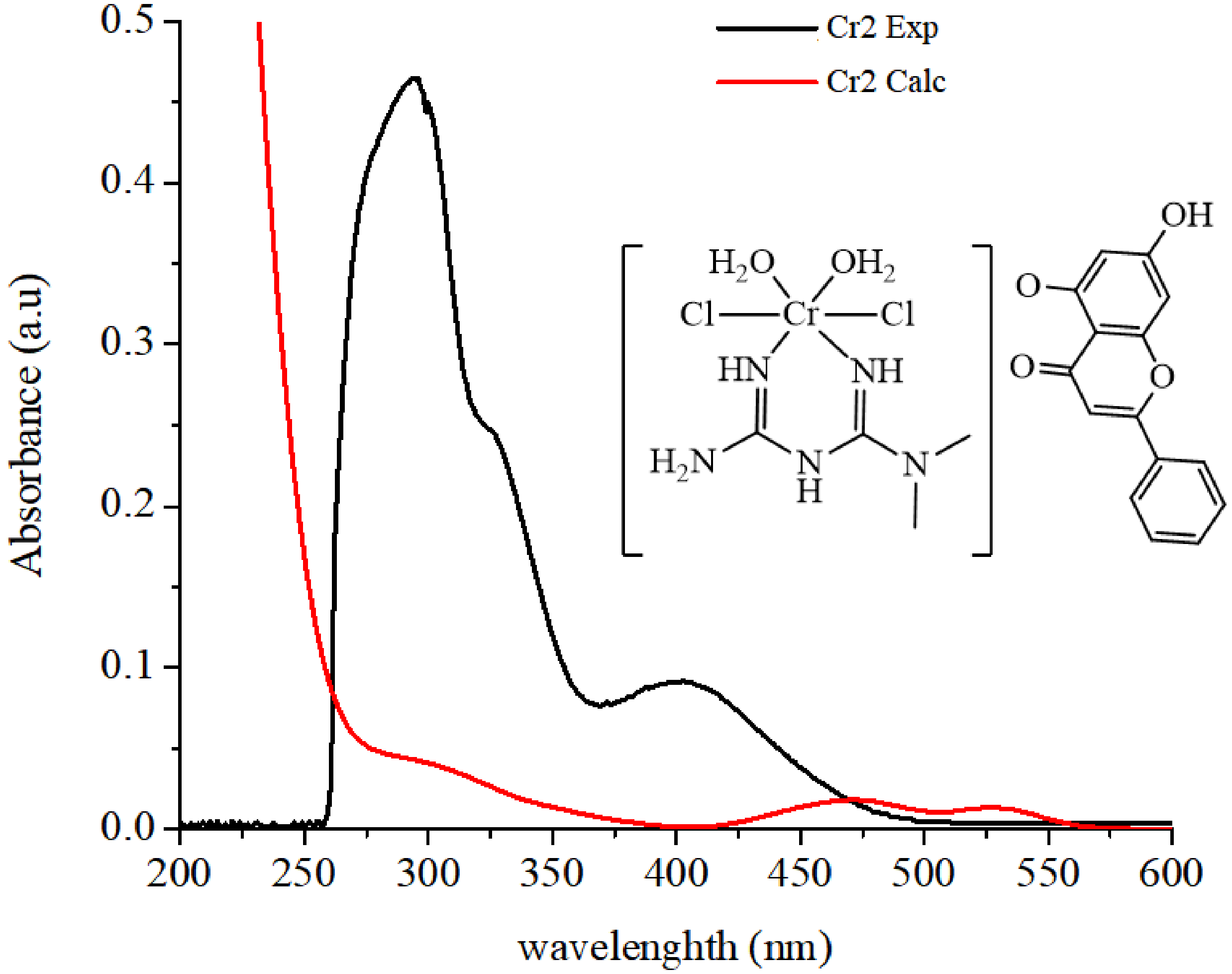

Figure S5. UV-Vis and TD-DFT calculated absorption spectrum for **Cr2** alternative structure

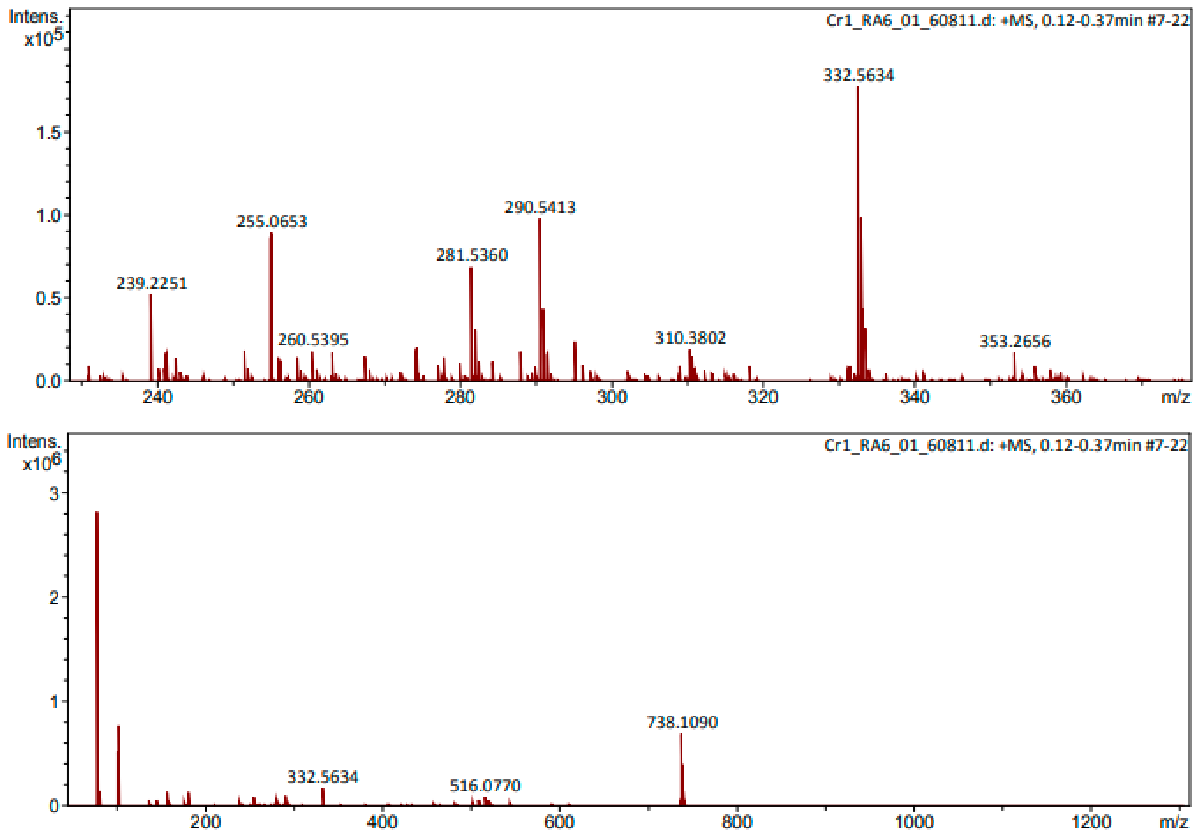

Figure S6. Mass spectrometric spectra of complex **Cr1**.

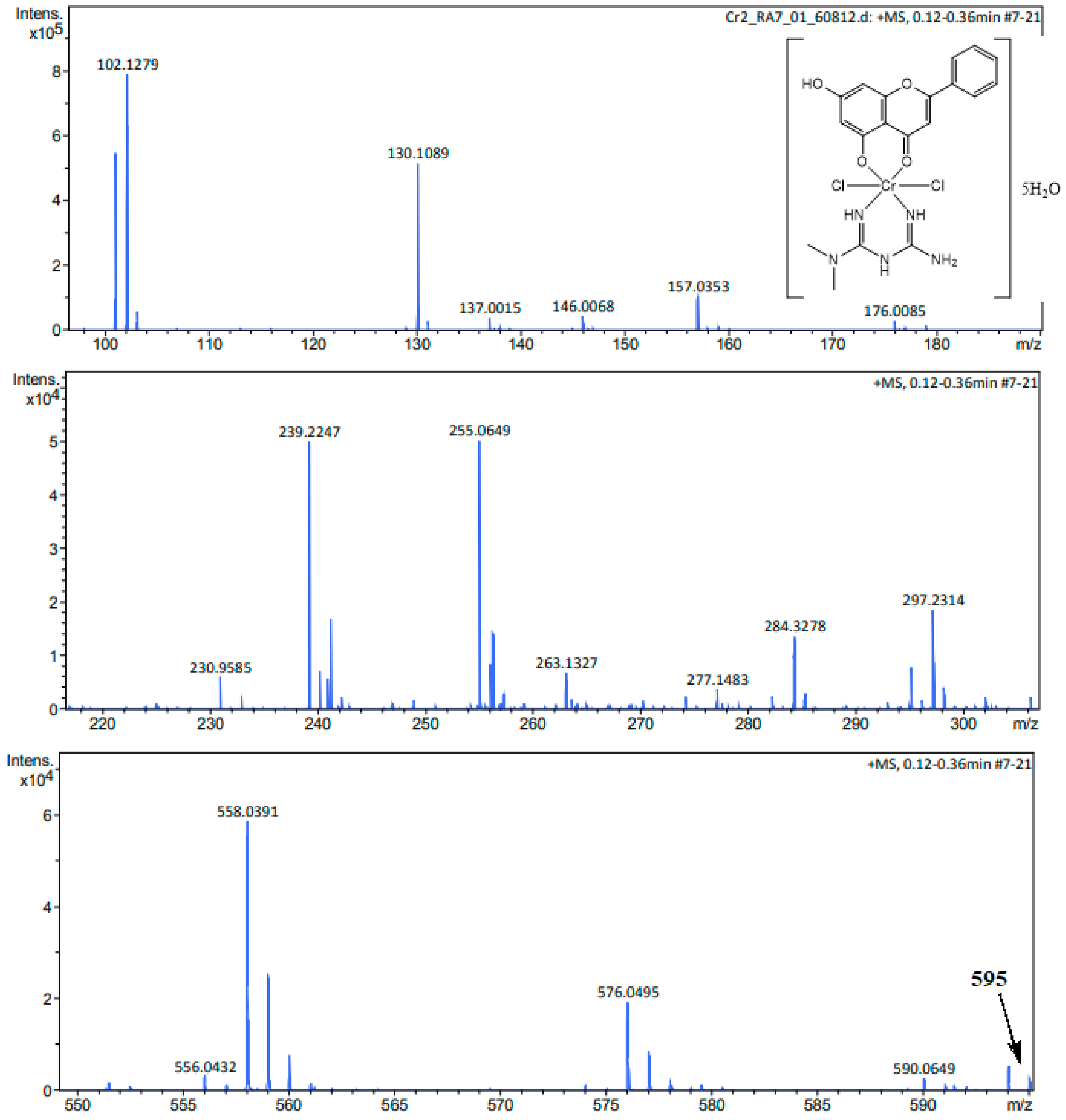

Figure S7. Mass spectrometric spectra of complex **Cr2.**

Table S1. Cytotoxicity measurements for percent cell viability of MCF-7 cell line

Conc Centration **(µM)**	**Cr1** repeats				**Cr2** repeats			
	n=1	n=2	n=3	Mean	n=1	n=2	n=3	Mean
0	100.00	100.00	100.00	100.00	100.00	100.00	100.00	100.00
3.125	103.43	95.04	81.49	93.32	94.74	104.06	95.05	97.95
6.25	87.77	66.18	61.43	71.79	93.44	109.53	101.37	101.45
12.5	62.08	8.74	7.07	25.96	87.40	102.13	83.66	91.06
25	9.90	0.38	2.65	4.31	81.85	96.04	75.14	84.35
50	1.40	2.75	3.80	2.65	73.98	93.72	42.19	69.96
100	1.61	4.38	13.61	6.53	43.31	79.13	65.86	62.76

Table S2. Antibacterial activity data of **Cr1** and **Cr2** complexes (MIZ (mm) ± SD)

Compound	Concentration (µM)	Gram-positive bacteria		Gram-negative bacteria	
		S. aureus ATCC25923	*S. pyogenes* (ATCC19615))	*E. coli* ATCC25922	*P.aeruginosa* ATCC27853
**Cr1**	500	8.17 ± 0.236	9.00 ± 0.816	8.00 ± 0.816	8.00 ± 0.707
	1000	10.00 ± 0.816	10.17 ± 0.624	10.00 ± 0.816	9.00 ± 0.816
**Cr2**	500	9.50 ± 0.408	10.00 ± 0.816	9.17 ± 0.624	9.33 ± 0.624
	1000	11.17 ± 0.624	12.00 ± 0.816	10.00 ± 0.707	10.00 ± 0.816
Ciprofloxacin	500	24.67 ± 0.236	23.00 ± 0.408	24.00 ± 0.816	22.333 ± 0.471

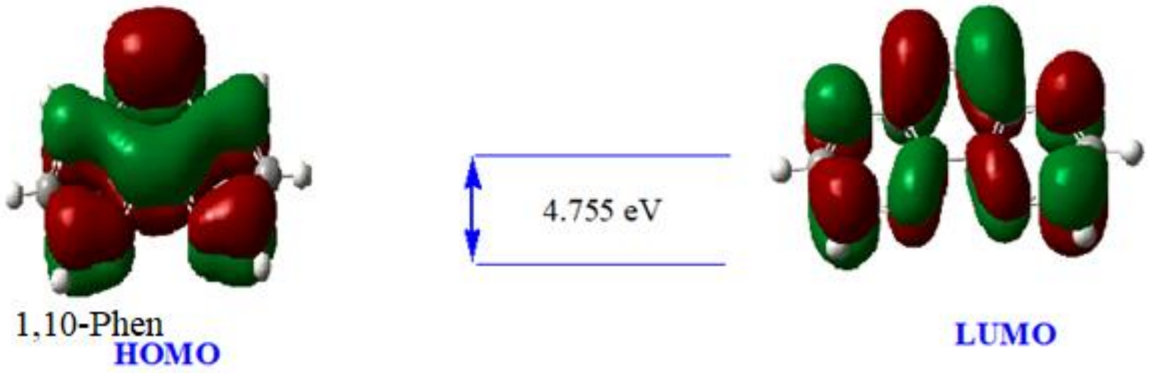

Figure S8. HOMO-LUMO distribution and band gap energy of 1,10-phenathroline

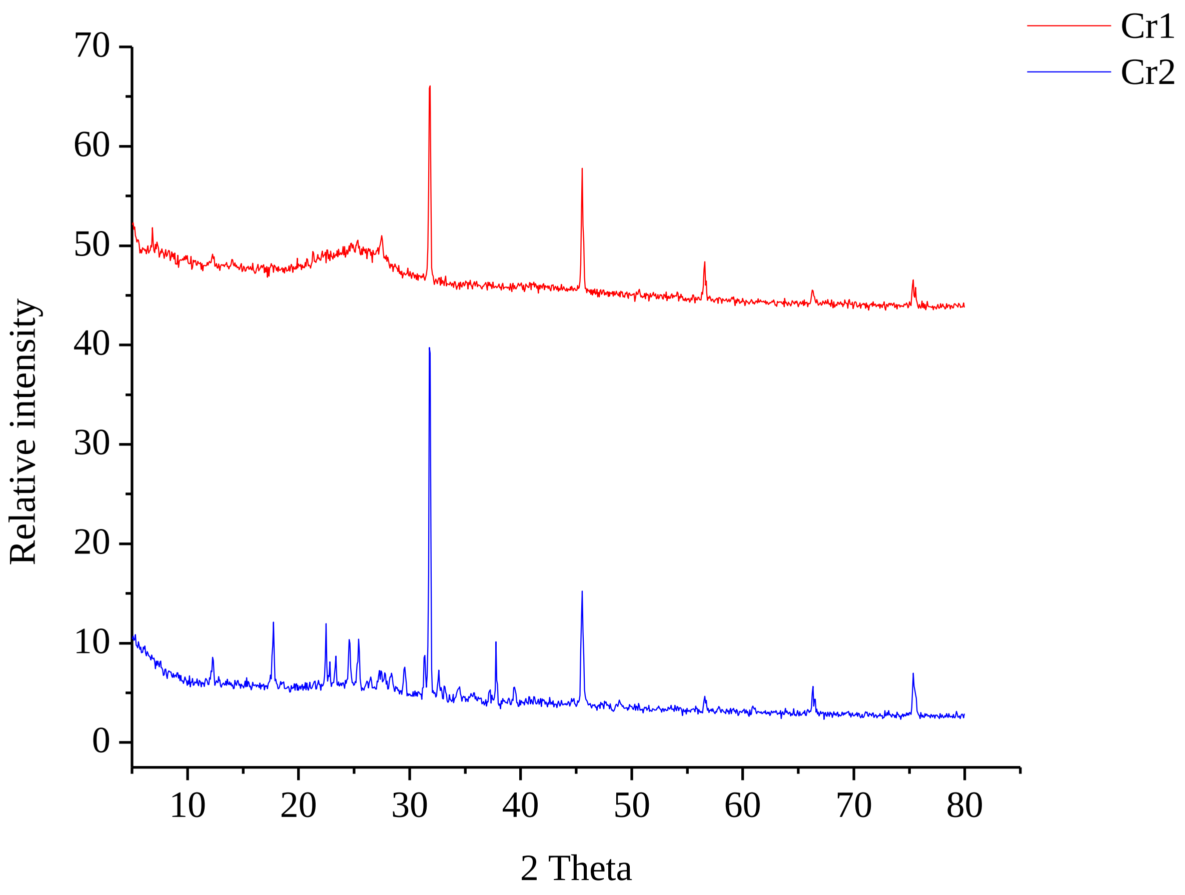

Figure S9. Powder x-ray diffraction patterns for **Cr1** and **Cr2** mixed ligand complexes

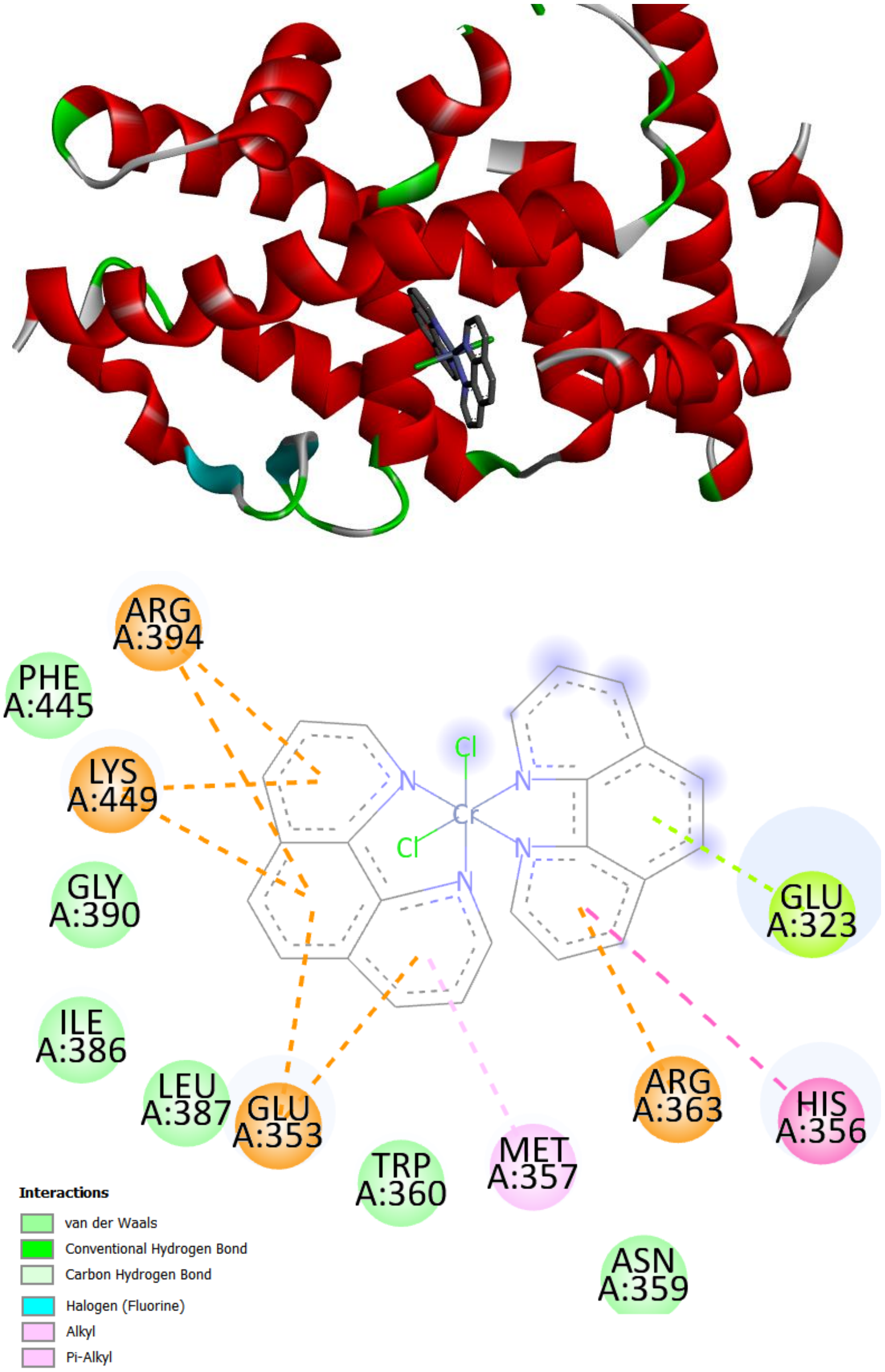

Figure S10. 3D and 2D representation of the interaction of **Cr1** in the binding pocket amino acid residue of ERa (ERα; PDB: 5GS4)

Table S3. Molecular docking scores and the corresponding prominent residual amino acid interactions of the complexes against *S. aureus* (PDB: 2w9h)

Cpds	RMSD	Binding energy (kcal/mol)	Inhibition constant (K_i)	H-bonding	van der Waals	π-alkyl/ π-ion
**Cr1**	0.81	-7.51	3.12 µM	-	His 23, Gln 19, Leu 54, Phe 92, Thr 46	Ser 49, Leu 20, Ile 50, Leu 28
**Cr2**	0.86	-8.82	0.30 µM	Leu 5, Phe 92	Leu 54, Asp 27, Val 31, Thr 46, Val 6, Phe 98. Trp 22, Ser 49 and Gln 19	Leu 28, Leu 20, Ala 7, Ile 4, Ile 50
**Cipro**	0.92	-8.66	0.45 µM	Asn 18, Asp 27	Gly 93, Ile 14, Gly 94, Val 6, Leu 5, Thr 111, Val 31, Leu 28, Thr 46, Ser 49, Lys 45	Leu 20, Ala 7, Phe 92

Table S4. Molecular docking scores and the corresponding prominent residual amino acid interactions of the complexes against *E. coli* (PDB: 6f86)

Cpds	RMSD	Binding energy (kcal/mol)	Inhibition constant (K_i)	H-bonding	van der Waals	π-alkyl/ π-ion
**Cr1**	0.45	-6.92	8.51 µM	-	Pro 79, Gly 77, Gly 75, Thr 265, Asp 73, Ala 47, Asp 49	Asn 46, Ile 78, Glu 50, Arg 50, Ile 94
**Cr2**	0.69	-7.51	2.78 µM	Gly 77, Asn 46	Val 44, Thr 165, Gly 75, Glu 50, Pro 79, Asp 49, Ile 94, Met 166	Gly 77, Val 167, Val 43, Ala 47, Asp 73, Val 71, Asn 46
**Cipro**	0.58	-6.19	28.85 µM	Arg 136, Gly 77, Asp 49	Thr 165, Asn 46	Arg 76, Glu 50, Pro 79, Ile 78

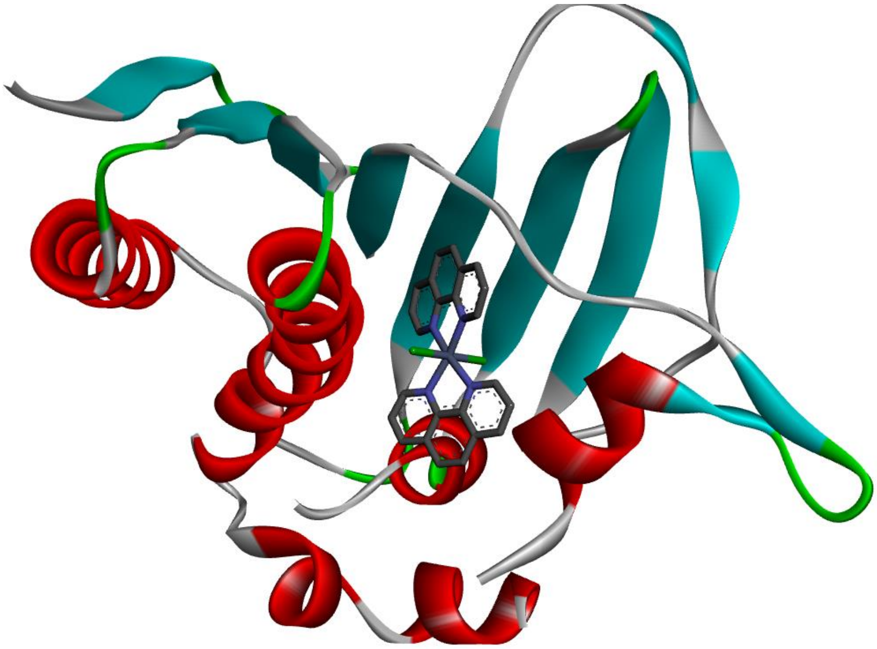

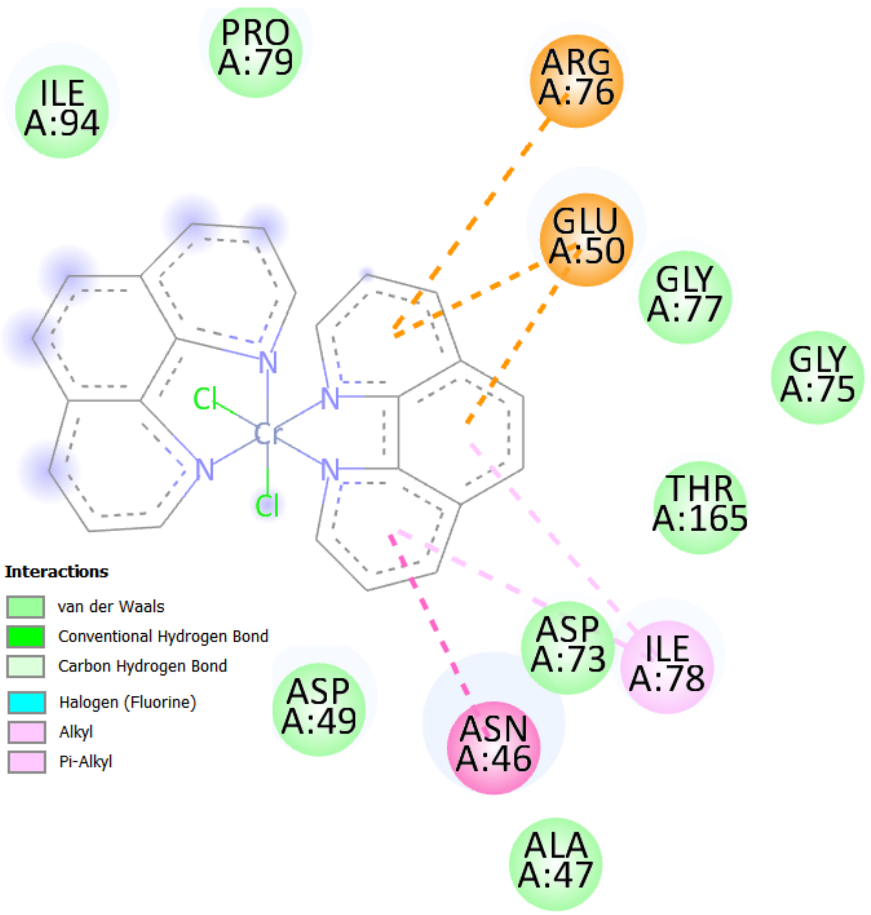

Figure S11. The binding interactions of **Cr1** against *E. coli* (PDB: 6F86)

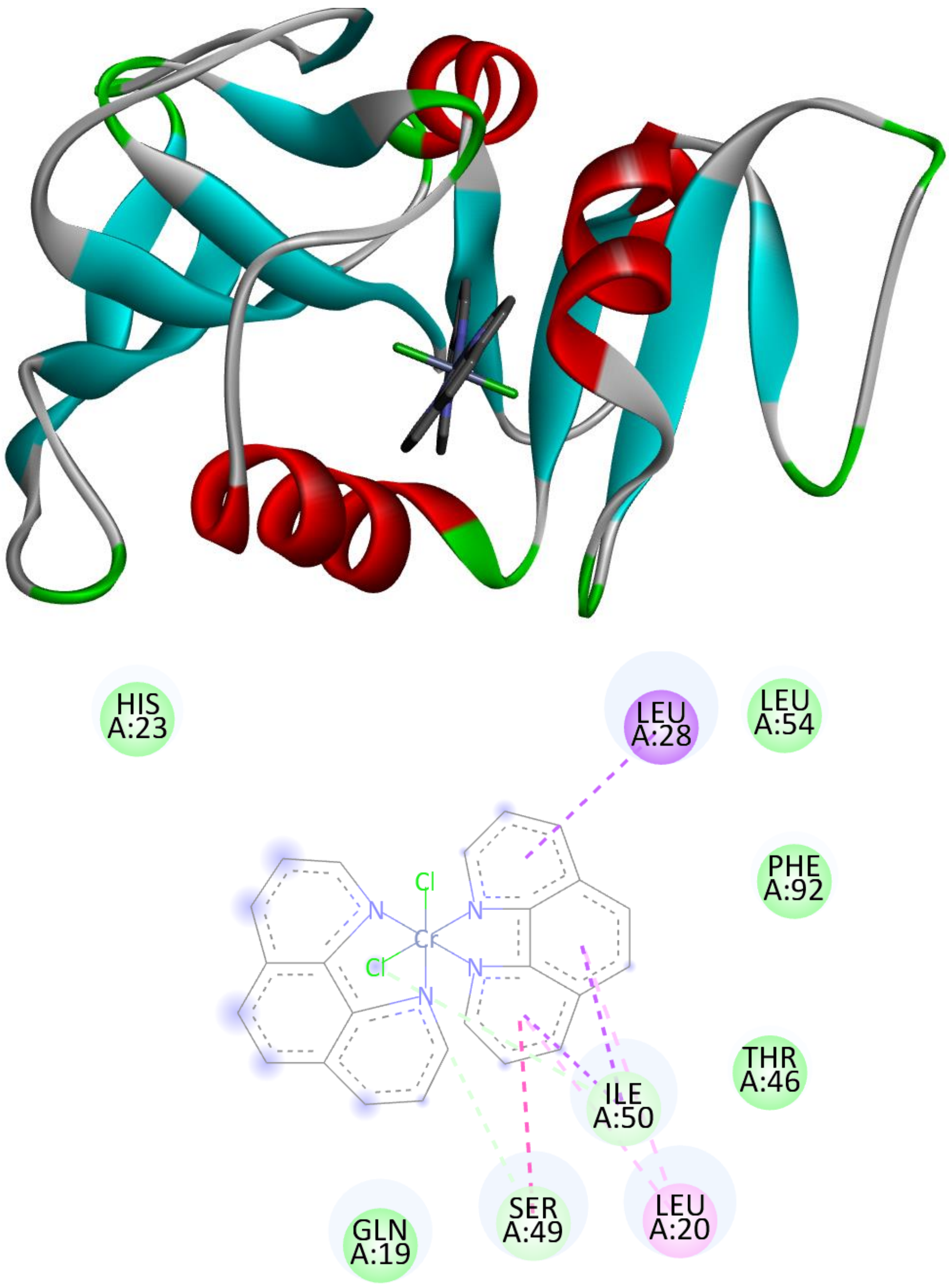

Figure S12. The binding interactions of **Cr1** against *S. aureus* (PDB: 2w9h)

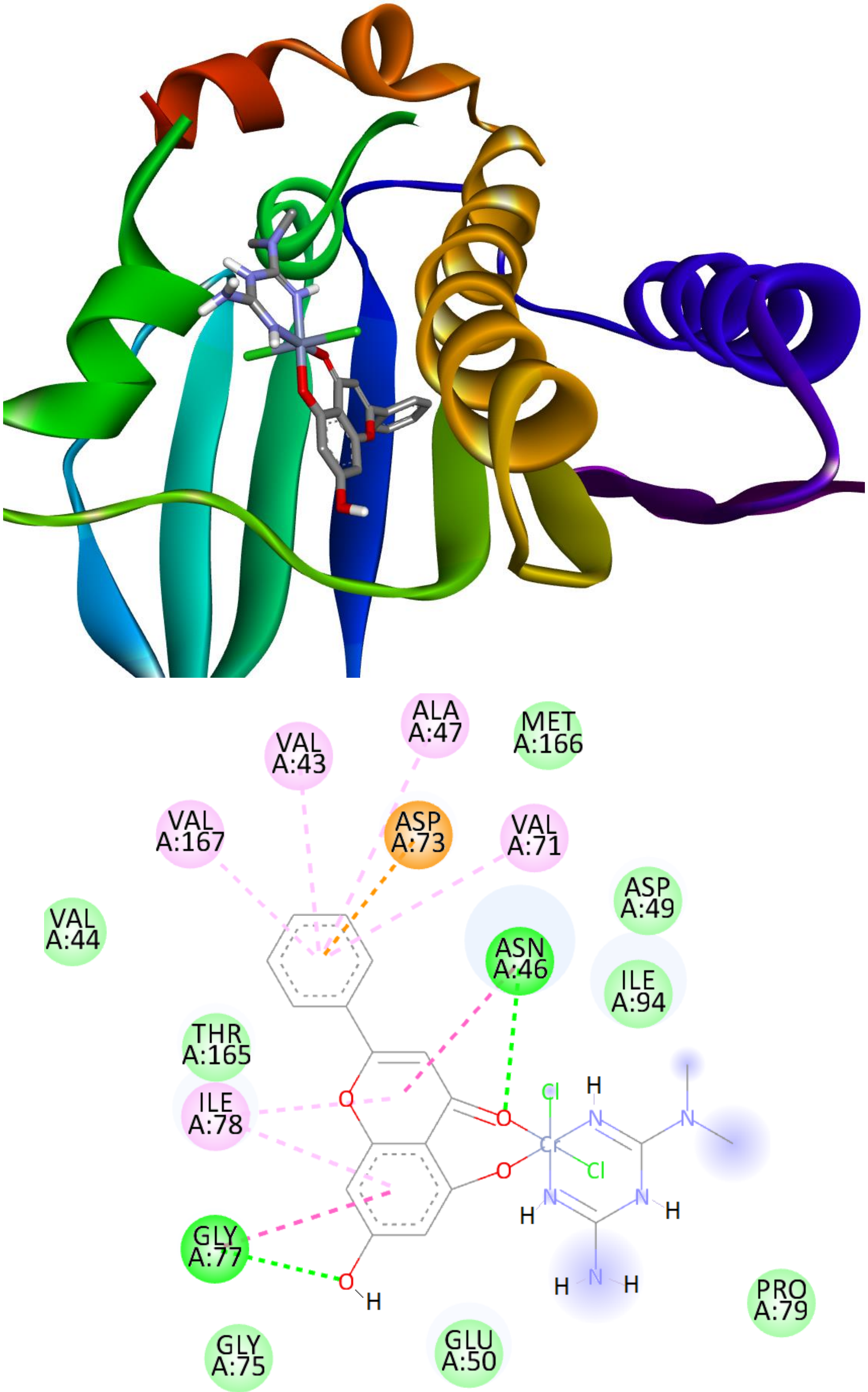

Figure S13. The binding interactions of **Cr2** against *E. coli* (PDB: 6F86)

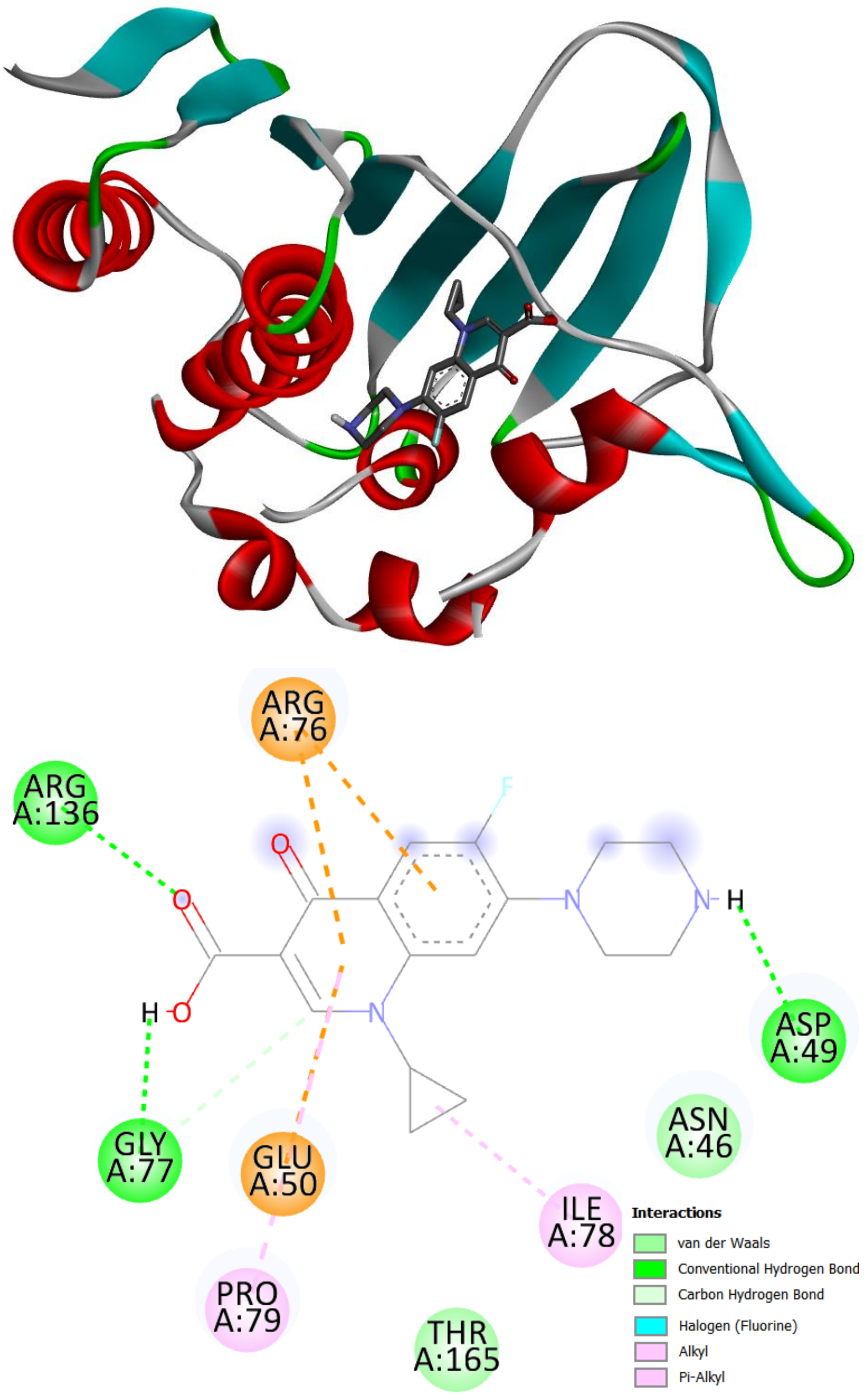

Figure S14. The binding interactions of **Ciprofloxacin** against *E. coli* (PDB: 6F86)

Table S5. B3LYP optimized geometry of Cr1

Cr	1.67226600	0.51097100	-1.61280300
C	5.82051100	-2.12026500	-1.15046800
C	5.39533200	-1.35902400	-0.07531900
C	4.19528100	-0.63506200	-0.17321600
N	3.42086300	-0.64003300	-1.26098800
C	3.86870000	-1.33690600	-2.34310900
C	5.05285100	-2.11664600	-2.33430600
C	3.08687100	-1.24164300	-3.53498600
C	3.44606100	-1.99739800	-4.67955800
C	4.63563000	-2.80328200	-4.63756700
C	5.41269800	-2.85158400	-3.51613700
N	2.00353600	-0.41640300	-3.49061200
C	1.20394800	-0.39730500	-4.55973200
C	1.46428400	-1.12608900	-5.73233300
C	2.59902400	-1.91570500	-5.80509400
C	-1.63773000	4.11144800	-2.25392300
C	-1.01320200	3.57813700	-3.36834300
C	-0.09598900	2.52595100	-3.20896600
N	0.21311300	1.99464300	-2.02361300
C	-0.35055100	2.55998100	-0.91932600
C	-1.30422900	3.60746000	-0.97893700
C	0.07789000	2.05682900	0.34721600
C	-0.51695300	2.54209400	1.53926900
C	-1.50418300	3.58338700	1.45214500
C	-1.87336500	4.10260100	0.24470100
N	1.05153800	1.10344100	0.32843200
C	1.38792200	0.54928800	1.49577900
C	0.84049100	0.94773400	2.72675800
C	-0.10171800	1.96146600	2.75638600
Cl	0.07446100	-1.32143300	-1.28091400
Cl	3.26195100	2.34478100	-1.96614900
H	6.73514700	-2.70308300	-1.09908300
H	5.96704500	-1.31353800	0.84482900
H	3.86989300	-0.02008400	0.65554800
H	4.90705000	-3.37076000	-5.52256000
H	6.31657100	-3.45295500	-3.49568500
H	0.30680200	0.20340100	-4.48748600
H	0.77306200	-1.05363100	-6.56454200
H	2.83518500	-2.47784600	-6.70356800
H	-2.36305400	4.91402800	-2.34721800
H	-1.22101600	3.95234200	-4.36453000
H	0.41276700	2.12022100	-4.07357300
H	-1.94737700	3.95370200	2.37170600
H	-2.61049700	4.89784400	0.18725400
H	2.10736500	-0.25831800	1.46374600

H	1.16990200	0.45694700	3.63571800
H	-0.53051900	2.30195500	3.69396700

Table S6. B3LYP Optimized geometry of Cr2

H	12.62042400	-19.84909100	-2.10512500
O	12.42758600	-10.61990900	0.84060100
C	10.33034400	-10.38359100	1.88776500
C	10.41625700	-8.98773900	1.72663400
C	9.48477500	-8.14987000	2.34106900
C	8.46144300	-8.69012200	3.12707500
C	8.37547000	-10.07684000	3.29907700
N	14.96599000	-19.36659600	-2.44827800
C	14.15270300	-18.56387200	-1.70969400
Cl	14.28996600	-16.03573300	1.61571100
C	9.30218900	-10.91935300	2.68651000
O	14.49795900	-14.54651000	-1.08904300
O	11.97082100	-14.61206800	0.15741300
Cr	13.24495500	-15.93536700	-0.57579300
C	11.94215700	-18.66749900	-0.56666700
H	9.45987900	-18.11164000	0.03779500
H	9.55740900	-7.07533200	2.20358500
H	8.93522200	-19.79645700	0.14617500
C	11.14972200	-12.59963300	0.98101400
C	13.36069500	-12.66359400	-0.08317700
H	10.66335200	-21.50651000	0.40668700
O	16.69262800	-10.41283200	-1.21879000
C	11.30239300	-11.26736000	1.22485200
N	14.47649300	-17.38082100	-1.28335200
N	12.89390600	-19.09746300	-1.48728400
Cl	12.14808600	-15.79697700	-2.73358900
H	15.90120000	-19.04278100	-2.65092400
H	7.73864200	-8.03631300	3.60581200
H	9.87205500	-19.08628600	1.47997900
H	10.24093100	-13.11422100	1.26166700
N	10.95830900	-19.55788000	-0.29014200
C	9.74415400	-19.10579200	0.39032200
H	16.40316000	-12.92198800	-1.65202200
H	7.59113700	-10.50246000	3.91739500
H	12.16501000	-21.28920700	-0.48695800
C	14.52295200	-10.48308500	-0.16437100
C	13.44069700	-11.27327200	0.18858800
C	15.58684000	-11.10790100	-0.83288400
C	12.17756000	-13.36324100	0.33214900
N	12.01771600	-17.46827700	-0.05805600
H	14.85599100	-20.37072100	-2.39976600

H	10.60882700	-21.36152700	-1.36411100
H	11.38920400	-17.29545800	0.71845900
C	14.47142500	-13.27973200	-0.77596800
C	15.56504900	-12.46992600	-1.13402500
H	11.20401700	-8.56425000	1.11406200
H	14.52805000	-9.42579700	0.07475300
H	15.45200400	-17.13223800	-1.41877100
H	9.23436100	-11.98958100	2.84920500
H	16.61974700	-9.48149600	-0.96443100
C	11.11146000	-21.00887900	-0.45667700
